# Complexity of αvβ6-integrin targeting RGD peptide trimers: emergence of non-specific binding by synergistic interaction†

**DOI:** 10.1039/d3md00365e

**Published:** 2023-09-28

**Authors:** Neil Gerard Quigley, Frauke Richter, Susanne Kossatz, Johannes Notni

**Affiliations:** a Institute of Pathology, School of Medicine, Technische Universität München Trogerstr. 18 D-81675 München Germany tum@notni.de; b Department of Nuclear Medicine, University Hospital Klinikum Rechts der Isar and Central Institute for Translational Cancer Research, (TranslaTUM), School of Medicine, Technische Universität München Munich Germany; c TRIMT GmbH Carl-Eschebach-Str. 7 D-01454 Radeberg Germany

## Abstract

Multimerization is an established strategy to design bioactive macromolecules with enhanced avidity, which has been widely employed to increase the target-specific binding and uptake of imaging probes and pharmaceuticals. However, the factors governing the general biodistribution of multimeric probes are less well understood but are nonetheless decisive for their clinical application. We found that regiospecific exchange of phenylalanine by tyrosine (chemically equivalent to addition of single oxygen atoms) can have an unexpected, dramatic impact on the *in vivo* behavior of gallium-68 labeled αvβ6-integrin binding peptides trimers. For example, introduction of one and two Tyr, equivalent to just 1 and 2 additional oxygens and molecular weight increases of 0.38% and 0.76% for our >4 kDa constructs, reduced non-specific liver uptake by 50% and 72%, respectively. The observed effect did not correlate to established polarity measures such as log *D*, and generally defies explanation by reductionist approaches. We conclude that multimers should be viewed not just as molecular combinations of peptides whose properties simply add up, but as whole entities with higher intrinsic complexity and thus a strong tendency to exhibit newly emerged properties that, on principle, cannot be predicted from the characteristics of the monomers used.

## Abstract 2

A single O atom at the right position halves liver uptake of αvβ6-integrin targeting RGD peptide trimers. As there is no correlation with polarity proxies, this is interpreted as an emergent phenomenon arising from loss of synergistic interaction.

## Graphical abstract

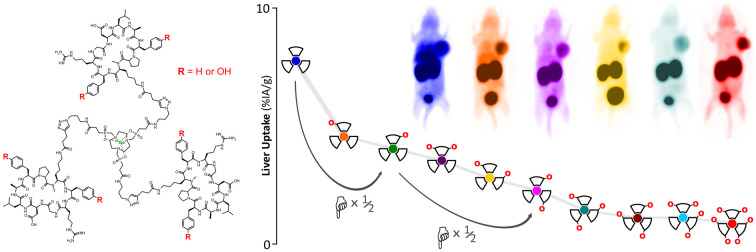

## Introduction

Multimers are macromolecular conjugates that link multiple biologics such as peptides, enzyme inhibitors, or antibodies in a covalent or non-covalent manner to enhance the pharmacological properties of the monovalent molecular units.^1^ Multivalency is typically exploited to enhance binding to a particular biological target (typically a receptor, transporter, or other cell surface protein) and has been widely used in the development of peptide-based radiopharmaceuticals.^2^ The underlying effect is referred to as avidity and is based on the assumption that the strength of the interactions is essentially a cumulative effect of all possible binding interactions.^3^ The number and strength of contributing ligand-target encounters depends on many variables, above all, the valency and geometry of the scaffold which determines multiplicity and distance between the conjugated functional monomers.^4,5^ In addition, linkers can be used to adjust the spatial distribution and motility of the individual ligands in a conjugate.^6^ The cumulative influence of these parameters is expressed by the degeneracy coefficient *Ω*_i_, which is used to formalize the thermodynamics of multimeric binding.^7^ The number of binding sites that a multimer can access simultaneously depends furthermore on the motility and clustering of cellular targets in the membrane.^8^ Multimers thus may exhibit altered target specificity or even bind to sites not recognized by monomers, as avidity not only enhances binding to the desired target but may also amplify unwanted or unknown weak interactions.^9^

However, multimerization is frequently also accompanied by substantial changes in general to *in vivo* properties and biodistribution, which can occur regardless of avidity or ligand-target binding thermodynamics.^10^ This is because multimerization inevitably increases molecular weight and often alters polarity, which may influence tissue penetration, elimination pathways and -kinetics, and plasma half-life.^11^ These interactions and effects are not easily described by simple models and formalisms, because one cannot focus on a few well-defined binding events but has to consider the agent's multifaceted interaction with the entire organism in its complexity. Böhmer *et al.* recently pointed out that multimeric contrast agents have not yet found their way into the clinical routine despite all efforts, and that a better understanding of this complexity is key to take this step.^8^ For this to happen, however, radiopharmaceutical development must break new ground, because both traditional and modern approaches to discovering new radiotracers predominantly consider interactions with the respective targets^12^ and thus might overlook off-target effects.

We previously used the triazacyclononane-triphosphinate (TRAP) chelator scaffold^13^ to elaborate highly symmetrical trimers^14^ of various tumor targeting motifs, such as prostate-specific membrane antigen (PSMA) inhibitors^15^ and ligands for the integrins αvβ3,^16^ α5β1,^17^ αvβ8,^18^ and αvβ6.^19^ After efficient labeling with the positron emitter gallium-68 (^68^Ga),^20^ these trimers were applied for positron emission tomography (PET) imaging of human tumor xenografts in rodent models. All integrin ligand trimers showed the desired increase in affinity and consistently improved *in vivo* imaging properties, namely higher tumor uptake, prolonged tumor retention, and higher target-to-background ratios.^21^

We subsequently specialized in radiopharmaceuticals targeting the heterodimeric transmembrane cell adhesion protein αvβ6-integrin which has recently attracted increasing attention because it is highly up-regulated in various malignant cancers,^22^ such as pancreatic ductal adenocarcinoma (PDAC)^23^ and oral squamous cell carcinoma (OSCC).^24^ Since it is furthermore a biomarker for fibrotic diseases^25,26^ and the Long Covid syndrome,^27,28^ αvβ6-integrin is a highly attractive target for biomedical imaging as well as targeted therapies.^29^ We focused our research on a class of cyclic nonapeptides described by Maltsev *et al.*,^30^ who identified the sequence c[FRGDLAFp(*N*Me)K] as a lead structure that specifically binds to αvβ6-integrin with sub-nanomolar affinity and is amenable to conjugation *via* the terminal amino group of the Lys side chain. Subsequent monomeric chelator conjugates of this peptide sequence failed at the preclinical stage because of inadequate target affinity and insufficient tumor uptake in murine xenograft models,^31^ whereas multimerization seemed to be a feasible way to achieve the desired *in vivo* performance. The corresponding TRAP-based trimer (in this work referred to as Y0) showed an increased target affinity but unfortunately proved unsuitable for clinical translation because of high non-specific uptake in many organs, particularly the liver.^32^ A modified sequence containing l-tyrosines (Y) instead of l-phenylalanines (F), c[YRGDLAYp(*N*Me)K], was next trimerized on the TRAP core.^33^ This ^68^Ga-labeled trimer, herein referred to as Y6, surprisingly lacked the undesired non-specific uptakes but retained a high affinity and selectivity for αvβ6-integrin, and thus was further developed as a clinical PET imaging agent.^34^ The radiopharmaceutical ^68^Ga-Trivehexin already proved its clinical potential for imaging of PDAC as well as head-and-neck squamous cell carcinoma (HNSCC).^35^

Since the chemical difference between Phe and Tyr consists in only one additional oxygen atom located between the *para*-carbon and -hydrogen atoms of the phenyl ring, the described replacement of 6 Phe in Y0 by Tyr in Y6 is equivalent to the concept that 6 additional hydroxyl groups have been attached to the first structure in place of hydrogens. We became intrigued by the fact that even minor structural differences, such as a few extra oxygen atoms or, likewise, hydroxyl groups in place of hydrogens, apparently did not significantly change overall avidity but had a surprisingly pronounced effect on overall biodistribution and excretion kinetics of macromolecules with a molecular weight exceeding 4 kDa.^33^ In particular, it did not appear obvious to us what influence the replacement of a single Phe, or specific groups of Phe, by Tyr would have on pharmacokinetics and general *in vivo* behavior of the trimers, and whether any such effects could be correlated with easily determinable *in vitro* parameters. We therefore systematically investigated the influence of the spatial distribution of Phe and Tyr units in ^68^Ga-labeled TRAP trimers of cyclo[XRGDLAXp(*N*Me)K] (X = F or Y) peptides on their *in vivo* behavior.

## Results

### Compound synthesis and characterization

**Fig. 1:**
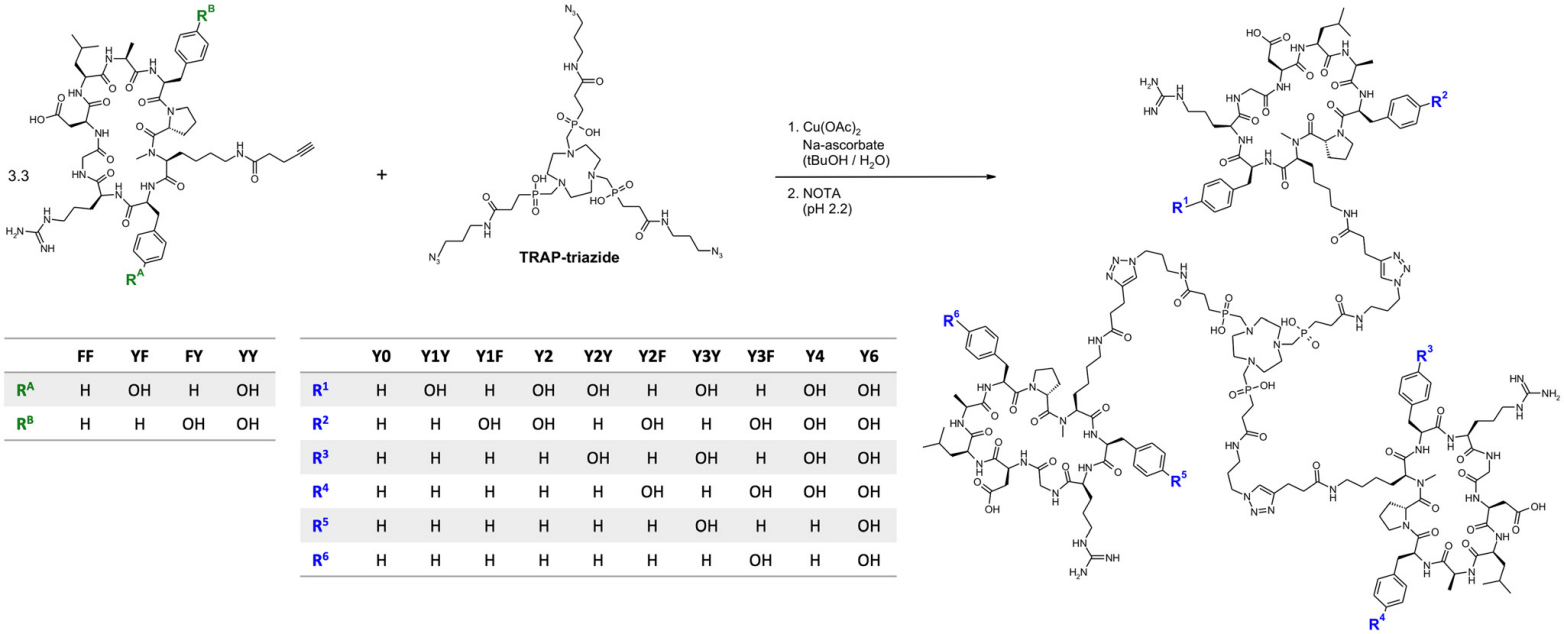
Synthesis of trimeric conjugates of αvβ6-integrin binding peptides. Synthesis of Y0, Y3Y, Y3F, and Y6 was done by reacting TRAP-triazide with the peptides FF, YF, FY, and YY, respectively. For the remaining conjugates, the CuAAC reaction was done using 1 : 1 mixtures of two different peptides (FF & YF, FF & FY, and FF & YY), leading to statistical mixtures of the respective hetero-conjugates which were separated by preparative HPLC.

Monomeric peptide building blocks bearing 4-pentynoic amides on the Lys side chains (see structures FF, FY, YF, and YY in Fig. 1) were synthesized according to a protocol that has previously been reported in detail.^33^ After assembly of the protected linear peptides by a solid-phase Fmoc approach, cyclisation in solution, and selective deprotection of the primary amine of Lys, 4-pentynoic acid was coupled to the Lys side chains of the four cyclopeptides cyclo[XR(Pbf)GD(*t*Bu)LAXp(*N*Me)K], wherein X = F or Y(*t*Bu), followed by acidic deprotection.

The integrin affinities of the monomers were determined using an established ELISA protocol,^36^ and expressed as 50% inhibitory concentrations (IC_50_, Table 1). Substitution of Phe by Tyr did not have a systematic effect. In comparison to FF, which has a subnanomolar αvβ6 affinity of 0.17 nM, the heterogeneous peptides YF and FY showed substantially diminished activities towards αvβ6-integrin, which was however partly restored for YY. The αvβ8-integrin affinity, which was 32 nM for FF, was not considerably affected by the Phe → Tyr exchange, with YF, FY and YY showing αvβ8-integrin affinities in the same range. Moreover, IC_50_ values >100 nM, such as those for α5β1- and αvβ3-integrin, indicate practically negligible binding and thus, are best interpreted as no activity. Hence, all four peptide variants are selective ligands for αvβ6-integrin, but FF and YY can nonetheless be considered more selective than YF and FY, owing to their substantially lower IC_50_ for αvβ6.

**Table 1:** Integrin affinities of alkyne-functionalized peptide monomers. Values are expressed as 50% inhibition constants (IC_50_), and were used to calculate the IC_50_-based selectivities. Data for FF and YY have been reported previously^33^ and are given for the objective of comparison

Compound	IC_50_ (95% confidence interval) [nM]				αvβ6-selectivity over		
	αvβ6	αvβ8	αvβ3	α5β1	αvβ8	αvβ3	α5β1
FF (ref. 33)	0.17 (0.09–0.33)	32 (20–51)	424 (270–670)	226 (115–193)	188	2494	1329
YF	3.3 (2.4–4.4)	23 (16–32)	630 (341–1162)	114 (82–158)	6.9	190	35
FY	2.8 (2.1–3.7)	37 (27–51)	732 (486–1104)	177 (138–227)	13.2	261	63
YY (ref. 33)	0.84 (0.56–1.2)	26 (19–37)	219 (88–540)	150 (116–193)	31	261	179

Peptide trimers with different spatial distribution of phenylalanines and tyrosines were then synthesized *via* CuAAC (Click Chemistry) conjugation followed by competitive demetallation.^37^ A threefold azide-functionalized TRAP core, referred to as TRAP-triazide,^38^ was reacted with the respective alkyne-functionalized peptide monomers (Fig. 1). The CuAAC reaction of TRAP-triazide was first performed using only one of the four alkyne-peptide building blocks at a time in order to firmly establish the procedure, resulting in the four canonical homotrimers. For the synthesis of the heterotrimers, we employed a combinatorial approach. CuAAC with equimolar amounts of of two different alkyne-functionalized peptides (FF + FY, FF + YF, and FF + YY) resulted in a statistical mixture of the respective four homo-and heterotrimers, which were subsequently separated by semi-preparative high performance liquid chromatography (HPLC). A total of 10 different trimeric conjugates containing zero, one, two, three, four, and six tyrosines at specific positions were thus obtained, referred to as Y0, Y1Y, Y1F, Y2, Y2Y, Y2F, Y3Y, Y3F, Y4, and Y6, depending on the number and position of tyrosines in the molecule (see Fig. 1; details of syntheses and yields are given in the Experimental section, Table 2).

**Table 2:** Trimer peptide composition and synthesis yields. Data for Y0 and Y6 (Trivehexin)^33^ have been previously reported and are included for the objective of comparison. The peptide subscript refers to the number of peptide constituents in the trimer molecule

Trimer	Y0	Y1Y	Y1F	Y2	Y2Y	Y2F	Y3Y	Y3F	Y4	Y6
Peptide composition	(FF)_3_	(FF)_2_, (YF)_1_	(FF)_2_, (FY)_1_	(FF)_2_, (YY)_1_	(FF)_1_, (YF)_2_	(FF)_1_, (FY)_2_	(YF)_3_	(FY)_3_	(FF)_1_, (YY)_2_	(YY)_3_
Yield	24.1%	7.1%	13.0%	12.3%	8.9%	16.0%	24.7%	42.8%	19.0%	54.2%

**Fig. 2:**
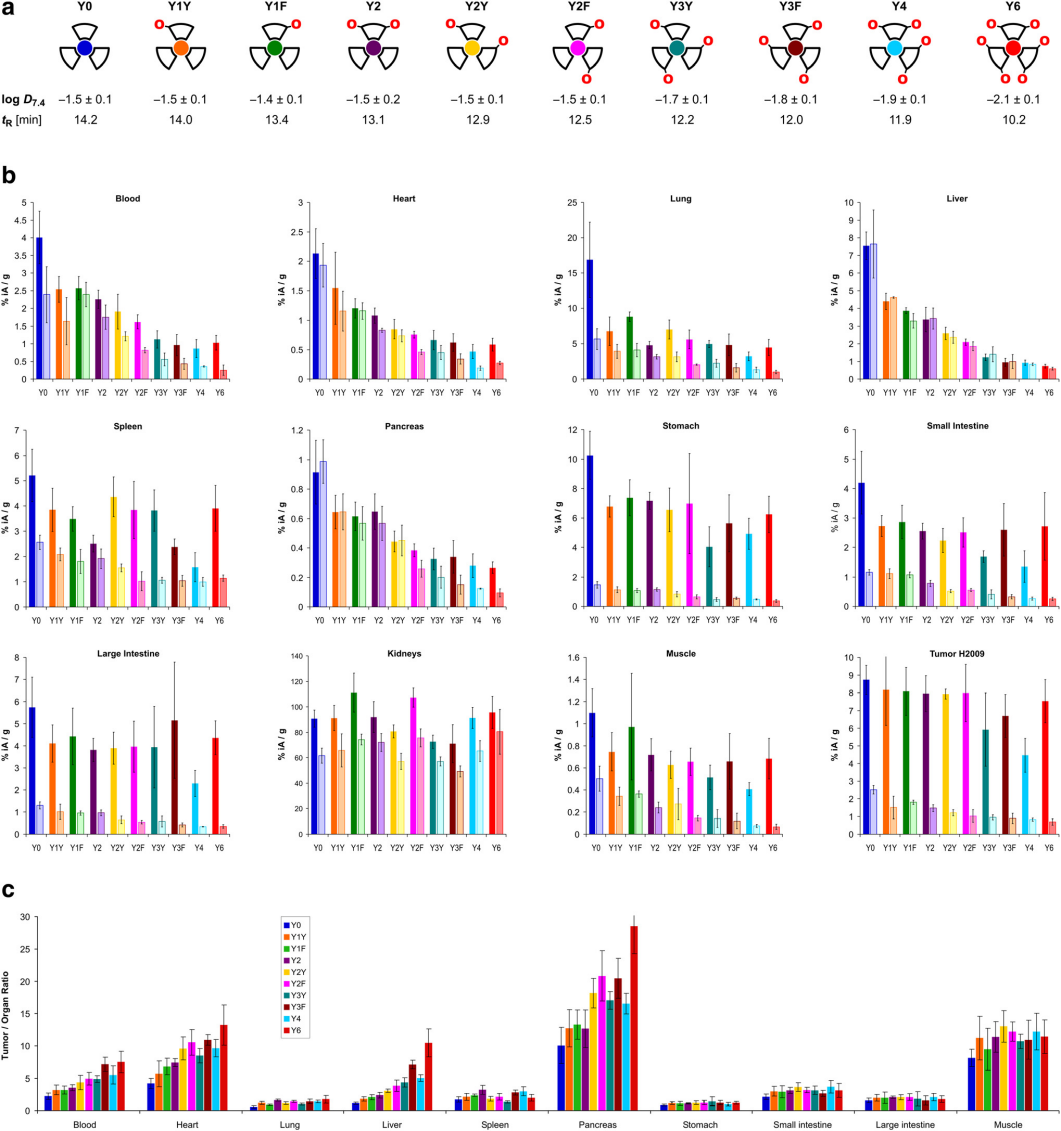
Biodistribution data for ^68^Ga-labeled peptide trimers, 90 min p.i.. a, Cartoon as aidé-memoire for the placement of tyrosines (symbolized by “O” letters, indicating that exchange of a Phe by a Tyr essentially translates to insertion of a single oxygen atom into the *para*-C–H bond of the aromatic ring); octanol–water (pH 7.4) distribution coefficients of the ^68^Ga labeled compounds (determined by shake-flask method using *n*-octanol as organic and PBS as aqueous phase, mean ± SD rounded to one decimal place, *n* = 8); and RP-HPLC retention times of the non-radioactive ^nat^Ga^3+^ complexes. b, Biodistribution in selected organs and tissues. Saturated bars indicate injected molar amounts of approx. 100 pmol (mean ± SD, *n* = 5); respective adjacent shaded bars of the same color show blockade (50 nmol unlabeled, administered 10 min before the radioactive compound, mean ± SD, *n* = 3). c, Tumor-to-organ ratios derived from biodistribution data (mean ± SD, *n* = 5). Numerical data for graphs shown in b and c, including the exact values of injected amounts, are provided in the ESI,† Tables S1–S10.

The trimers were labeled with ^68^Ga for biodistribution and small-animal PET experiments employing a fully automated method.^39^ Non-radioactive reference compounds were obtained by complexation of equimolar amounts of ^nat^Ga^3+^ in form of the aq. chloride solution.^39^ The polarity of the ^68^Ga or ^nat^Ga^3+^ containing conjugates, respectively, was assessed by two established proxies, namely the *n*-octanol/water distribution coefficient at pH 7.4, log *D*_7.4_, and the retention time *t*_R_ on a reverse-phase HPLC column with a suitable liquid-phase gradient (10–50% acetonitrile in water with 0.1% trifluoroacetic acid) (see Fig. 2a). Interestingly, the log *D*_7.4_ of the six trimers with zero (Y0), one (Y1Y and Y1F), and two tyrosines (Y2, Y2Y, Y2F) is found in a narrow range from −1.4 to −1.5 and must be considered similar, with respect to the error margins. A measurable decrease of the log *D*_7.4_ was only observed for ≥3 Tyr. The HPLC method, however, showed different *t*_R_ for all conjugates, and was not only able to discriminate between different numbers of tyrosines, but also between conjugates containing the same number of tyrosines located at different positions (*e.g.*, Y1Y*vs.*Y1F, with *t*_R_ of 14.0 and 13.4 min, respectively; or Y2*vs.*Y2Y*vs.*Y2F, with *t*_R_ of 13.1, 12.9, and 12.5 min, respectively).

### *In vivo* biodistribution and PET imaging

The biodistribution of all ^68^Ga-labeled peptide trimers was investigated in severe combined immunodeficiency (SCID) mice bearing subcutaneous xenografts of the αvβ6-integrin expressing human lung adenocarcinoma cell line H2009 on their right shoulders. Fig. 2b shows that the extent of non-specific binding, reflected by the fraction of non-blockable uptake, is generally varying between organs and tissues. For example, uptakes in liver and pancreas are completely non-specific, which corresponds to the ITGB6 negativity of these organs.^32^ In contrast, the stomach, intestine, lung, and tumor tissues show high fractions of blockable uptake corresponding to known physiological ITGB6 expression in mice or the H2009 tumor tissue.^32^ The generally low uptake in muscle was also reduced by blockade by a certain extent, giving a hint on a very low ITGB6 expression in this tissue. Reduction of the blood uptake upon blockade can, however, not be explained at present.

**Fig. 3:**
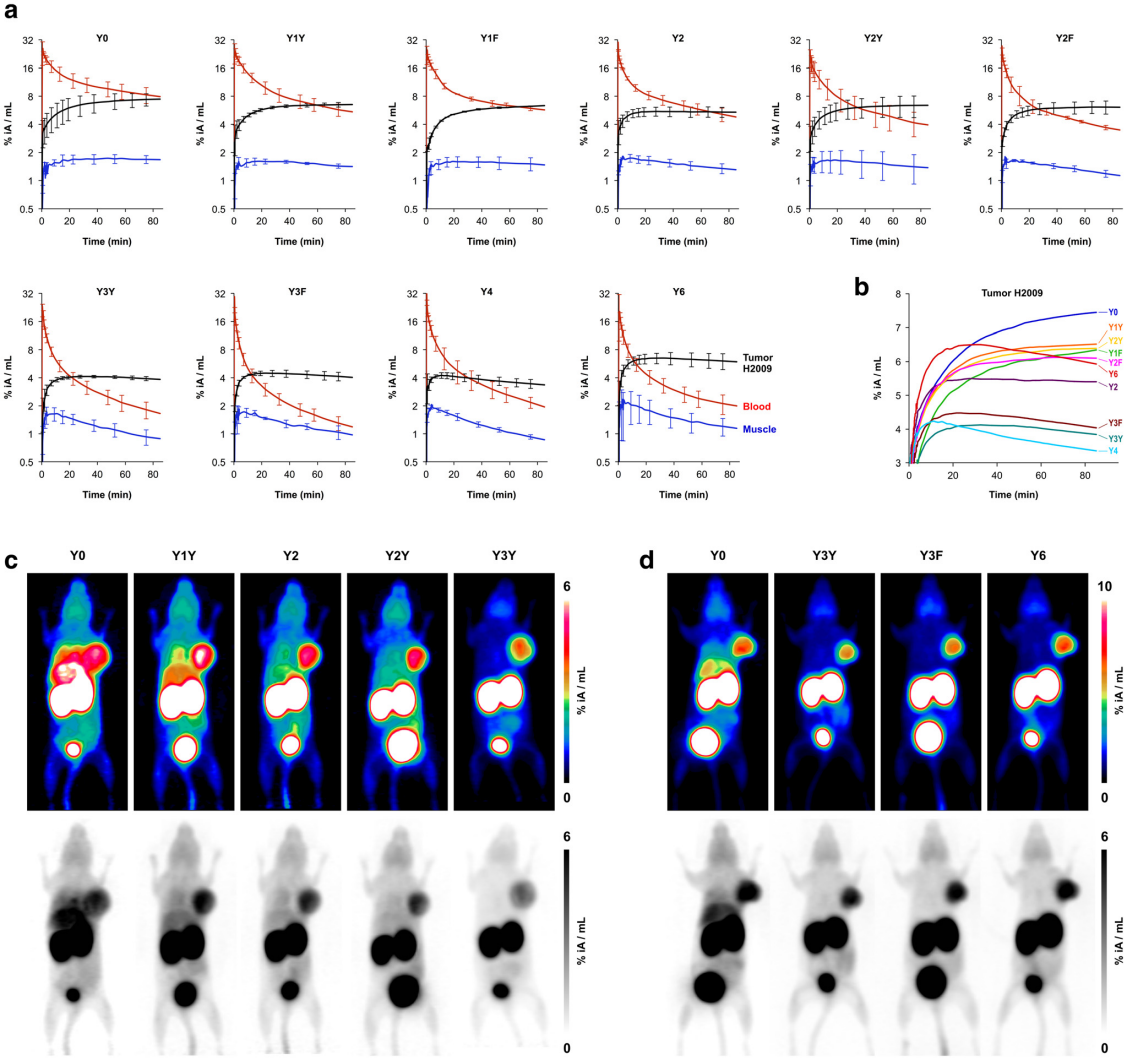
Positron emission tomography (PET) of severe combined immunodeficient (SCID) mice bearing subcutaneous H2009 (human lung adenocarcinoma) xenografts on the right shoulder. Dynamic scans for determination of biokinetics (a and b) were recorded in list mode and reconstructed in multiple frames with increasing time spans. Static images (c and d) were recorded over 20 min, 75 min after tracer administration, and reconstructed as single frames using the OSEM3D algorithm. The same scans are depicted as maximum intensity projections (MIP) employing both a color scheme and in grayscale (aligned vertically). a, Time-activity curves showing tracer elimination from the blood pool as well as accumulation in tumor and non-target tissue (skeletal muscle) (mean ± SD, *n* = 3). b, Comparison of tracer kinetics in tumor tissue (average of *n* = 3, error bars omitted for clarity). c, Comparison of PET images obtained with trimers containing the “*YX*” sequence motif in the same animal (subsequent tracer administrations and PET scans with an interval of 6–24 h). d, Comparison of PET images using trimers with a symmetrical conjugation pattern in the same animal. Numerical data for graphs shown in a and b are provided as ESI,† Tables S11–S20.

The Phe → Tyr exchange had a strong influence on biodistribution (Fig. 2) as well as dynamic and static PET imaging (Fig. 3). Even the introduction of a single Tyr significantly reduced non-specific uptake at 90 min p.i., with the most pronounced changes seen in liver, heart, and pancreas (Fig. 2b). Progressive Phe → Tyr exchange led to accelerated clearance from the blood pool (Fig. 3a), which was consistent with lower blood activity measured after 90 min for the tyrosine-rich derivatives (Fig. 2b). Interestingly, this pattern was not reflected in the tumor tissue. Whereas tumor uptakes remained largely constant for the six trimers comprising zero to two Tyr, a reduction was observed for three and four Tyr in Y3Y, Y3F, and Y4, which somewhat corresponds to the pattern observed for the log *D*_7.4_ values. However, the original tumor uptake level was restored for Y6. A simple reference to polarity therefore cannot explain our observations. Interestingly, the tumor time-activity curves of derivatives with ≥3 Tyr showed a maximum within the monitored time period of 90 min (see Fig. 3b), which appears to be mainly associated with compound polarity. The consequences for tumor visualization and overall imaging performance were illustrated by two different series of intra-individual PET imaging, where the same animal underwent PET imaging after the injection of different ligands on consecutive days, allowing us to directly compare tumor uptake and biodistribution (Fig. 3c and d). Fig. 3c demonstrates the strong influence of one and two Tyr on background (organ) activity, leaving no doubt that the pronounced liver and lung uptake of the Phe-only trimer Y0 compromises the tumor imaging capabilities. There were notable differences between individual animals, illustrated by the fact that the animal displayed in Fig. 3c generally exhibited an overall lower tumor uptake than the animal in Fig. 3d across the different radiotracers. However, such differences between individual animals are commonly observed as a result of the interindividual heterogeneity of H2009 xenograft growth, resulting in variations of αvβ6-integrin expression density in the H2009 tumors. Nonetheless, Fig. 3d demonstrates that Y6 offered the best combination of lowest background with high tumor uptake, which was also reflected in the most favorable tumor-to-organ ratios observed for this compound (Fig. 2c).

## Discussion

### Structure, polarity, and biodistribution

The *n*-octanol/water distribution coefficient at physiological pH, expressed as its decadic logarithm log *D*_7.4_, is one of the standard polarity proxies that are used to predict a molecule's distribution in fatty *vs.* aqueous compartments of tissues, or to assess its potential to passively cross lipid bilayers and other physiological separators, such as the blood–brain-barrier.^40^ The polarity of structurally related compounds can furthermore be compared by determining their retention times, *t*_R_, in a given chromatography or HPLC setup.^41^ According to textbooks of pharmaceutical sciences, the overall polarity of a molecule widely determines its pharmacokinetics and biodistribution. Non-polar molecules are usually more prone to be absorbed by liver tissue and/or excreted though the hepatobiliary pathway than polar compounds which are ordinarily excreted renally. Nevertheless, a quantitative threshold for liver absorption and hepatobiliary excretion, such as a fixed cutoff for the log *D*_7.4_ value, cannot be given since other structural parameters determine the pharmacokinetics as well. Decreasing the lipophilicity of a given structure by introduction of polar moieties nonetheless usually results in a lower liver absorption and a lower fraction of hepatobiliary excretion. This approach can not only be exploited to fine-tune the pharmacokinetics of drugs and drug-like substances, but it is also the physiological way in which higher animals regulate the excretion of lipophilic substances, namely, by oxidative metabolization *via* the cytochrome (CYP) enzyme family. Along these lines of thought, we set out to mitigate the high and unwanted liver uptake of the integrin αvβ6-targeting peptide multimer Y0 by increasing the hydrophilicity of the entire molecule with polar modifications, namely by replacing all Phe by Tyr. This approach was successful and resulted in the discovery of Y6 (^68^Ga-Trivehexin).^33^

Concerning the stepwise substitution described in the present study, we assumed that the number of Phe → Tyr exchanges should determine the extent of liver absorption, and furthermore be correlated to polarity measures such as log *D*_7.4_ or *t*_R_. Phe is considered a non-polar amino acid, and the phenyl moiety in its side chain may be causative for prolonged blood pool retention due to enhanced albumin binding, as well as a higher absorption. We expected, therefore, that regardless of the actual number, the mere presence of phenyl substituents in our trimer library should essentially result in a biodistribution profile which suggests the trimer is lipophilic, *i.e.*, the step from zero (Y6) to two (Y4) phenyl units should have a greater effect on liver uptake and blood protein binding than six phenyls (Y0) *vs.* five (Y1Y, Y1F) or four (Y2, Y2Y, Y2F).

To our surprise, we observed the opposite. The replacement of two Tyr by Phe in Y6, resulting in Y4, did not significantly change the uptakes in organs with predominantly non-specific uptakes, particularly the liver but also blood, heart, and pancreas (Fig. 2b). On the other hand, introducing one or two Tyr into the all-Phe conjugate Y0 had a dramatic effect on non-specific organ uptake of the respective radiolabeled compounds. It is worthwhile to examine this in more detail, with a closer look at the structural changes at the atomic level. The difference between the zero-Tyr conjugate Y0 and the mono-Tyr derivative Y1F consists in a single additional oxygen atom, equivalent to a +0.38% increase in molecular weight (MW). Nonetheless, the liver uptake of the ^68^Ga labeled Y1F was only 50% of ^68^Ga-labeled Y0 (Fig. 2b). Likewise, the MW difference between Y0 and Y2F is 32 Da or +0.76%, yet the liver uptake of ^68^Ga-labeled Y2F was about 72% lower than that of its Y0 congener. We deem it very remarkable that such small changes can have such a big impact. In the field of radioactive probe development, moderate structural changes, such as introduction of radioactive fluorine atoms or fluorinated prosthetic groups like [^18^F]fluoroethyl or [^18^F]fluorobenzoyl, are often unavoidable but frequently found to be of no concern regarding pharmacokinetics. Even the conjugation of substantially different radiometal chelates with MW exceeding 500 Da to a given peptide sometimes has only a small influence on the *in vivo* behavior. The highly sensitive response of the biodistribution of the present trimers to minuscule structural changes readily suggests that a more complex mechanism than a deterministic structure–activity relationship may be at work here.

**Fig. 4:**
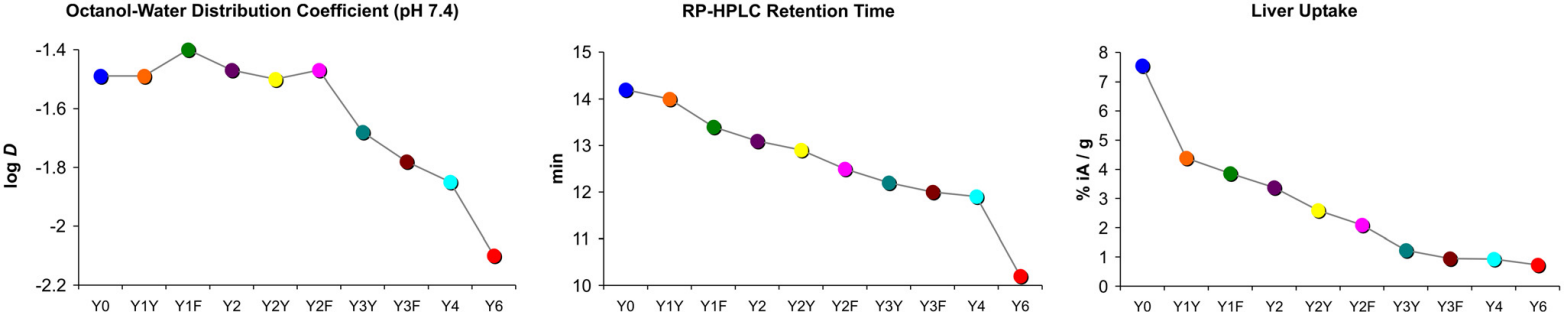
Comparison of trends in polarity proxies and liver uptakes. Plots were derived from data presented in Fig. 2. Connecting lines between data points are intended only to visualize trends and do not indicate a functional correlation.

Another surprising finding was that the log *D*_7.4_ did not show the expected pattern. Some of the measured values did not correspond to the basic principle that polar moieties introduced by (formal) oxidation increase hydrophilicity. Fig. 4 shows that the log *D*_7.4_ did not significantly change with the introduction of up to two hydroxyl groups. A considerable move was seen first for the step from two Tyr to three Tyr, as well as for subsequent replacements. On the other hand, the retention time on a reverse-phase HPLC column, *t*_R_, showed a trending pattern which was more in accordance with expectations. A gradual decrease of *t*_R_ with increasing Phe → Tyr substitutions was observed. Unlike the log *D*_7.4_, the *t*_R_ value furthermore provided a clear distinction between all regioisomers and reproduced the same order of the compounds that was observed for the liver uptakes. These findings are in line with previously reported inconsistencies between the two methods.^41^

However, with regard to the original question of the influence of polar modifications on liver uptake, herein discussed representatively for the other tissues with non-specific uptake, we found that none of the polarity measures was able to quantitatively predict changes of this parameter. Fig. 4 shows that the substantial increase of both log *D*_7.4_ and *t*_R_ for the Y6 → Y4 transition does not relate to a notable change of this value. On the other hand, the highly relevant and practically important reduction of liver uptake associated with the Y0 → Y1X transition is not adequately reflected by changes of *t*_R_ and particularly of log *D*_7.4_. These findings confirm that polarity proxies sometimes are of limited value in guiding drug candidate selection in pharmaceutical research processes,^41^ underscoring the need to include more sophisticated experiments at an early stage to avoid overlooking such patterns and inadvertently excluding promising candidates from a development pipeline.^40^

### Complexity, synergy, and emergence

At this point it must be noted that the observed phenomena, most succinctly illustrated by the finding that the addition of a single oxygen atom into a molecule larger than 4 kDa can reduce non-specific liver uptake by half (Y0 → Y1F), defy explanation based on one of the aforementioned cause-and-effect relationships established in pharmaceutical science. As stated above, one would have expected that the largest impact of all Tyr → Phe exchanges should be observed for newly introduced Phe, *e.g.*, zero Phe to two Phe (Y6 → Y4), and not for five Phe to six Phe (*e.g.*, Y1Y → Y0). An introduction of one or a few Phe into Y6 did not increase blood pool retention or liver uptake as expected. Likewise, putting one or two Tyr into Y0 did not change hydrophilicity according to the log *D*_7.4_, but nonetheless removed a large fraction of non-specific uptake in the liver and elsewhere.

These seemingly contradictory findings call for a fundamentally different explanatory approach. The central question that needs to be asked is obviously not what to *add* to the molecules to achieve the observed effect, but what to *subtract*. If we look at it from this point of view, we notice that transitions from six Phe to five Phe (*e.g.*, Y0 → Y1Y) have the largest impact, followed by the next Phe → Tyr substitutions, *e.g.*, Y1Y → Y2Y. In short, not the *addition* of anything, but *subtraction* of the first Phe from Y0 removes most of its non-specific uptake. This finding readily passes a test for the presence of synergy, which, according to P.A. Corning,^42^ was first suggested by Aristotle in the *Metaphysics*: remove a part and observe the consequences. If an observed phenomenon is gone afterwards, it must have been the product of a synergistic interaction. In such cases, the phenomenon is referred to as an emergent one. In many cases, the synergy might not be complete; hence one might have to remove more than one part to fully remove the synergy (according to Corning, call it synergy minus *n*).^42^ Along this line of thought, we assume that the six Phe in Y0 engage in a form of synergistic interaction, leading to the emergence of disproportionally high non-specific uptake, particularly in the liver. This emergent phenomenon disappears as the synergy is gradually destroyed by replacing phenylalanines by tyrosines, the first replacement naturally having the greatest impact.

The obvious question of which specific parts of the molecule interact in this process, and how exactly they do so, is admittedly not easy to answer. We assume that the high symmetry of the trimers might play a central role. A similar pattern was observed earlier during trimerization of a neurotensin-binding peptide, where the symmetrical TRAP trimer (log *D* = −3.7) showed a surprisingly high non-specific liver uptake (11% IA/g) that could not be inferred from the data of the monomer (log *D* = −4.1; 0.3% IA/g).^10^ Further investigations on comparable trimers based on scaffolds with a lower or no symmetry could help to clarify this point. Our current data nonetheless provide evidence that the synergistic interaction occurs primarily between Phe located on different peptides. Fig. 4 shows that, for example, the transitions from Y1Y and Y1F to Y2 have a much lower effect on liver uptake than to the transition between Y2 to the species Y2Y and Y2F wherein the Phe are distributed to different peptide moieties. This is not surprising, however, because if the observed emergent phenomena were indeed due to multimerization, *i.e.*, the presence of multiple peptides in the same macromolecule, then multiple modifications should show greater effects if they are not made to the same peptide but to different monomeric units. In other words, a synergistic interaction between individual peptides is apparently destroyed more efficiently when multiple peptides are modified rather than just one.

### Peptide trimers are whole entities

The data and results of this work have convinced us that the trimers presented herein, and multimers in general, must be viewed as holistic entities and consequently need to be perceived and studied with a view toward the whole rather than the parts. To best convey this message, we have chosen the compound codes in this work to reflect the number and position of tyrosines in the radiotracers as wholes, rather than classifying and discussing the trimers according to the monomers they comprise of. We furthermore like to underscore that the phenomena described herein could not have been anticipated due to their emergent nature, *i.e.*, the specific *in vivo* properties of any of the 10 investigated trimers could not have been predicted by cumulative consideration of the characteristics of the different monomeric peptides used. Any such reductionist approach, whose predictive power is primarily based on summarizing parameters of the components, is prone to overlook newly acquired properties of multimers that may prove critical for *in vivo* or medical applications. We therefore hold the view that predominantly deterministic concepts, such as step-by-step guides for the construction of multimeric imaging probes,^8^ are not capable to predict, nor circumvent, emergent properties of multimers resulting from unexpected synergistic interaction of their components.

## Conclusion

This study demonstrated that in the field of multimeric peptides for *in vivo* applications, the complexity of the interaction of the multimers with the living organism can play a crucial role and places severe limits on the predictive power of reductionist design approaches of any kind. We conclude that peptide multimers should be perceived not just as combinations of a number of peptides whose properties simply add up, but as whole entities with higher intrinsic complexity and thus a strong tendency to exhibit newly emerged properties. It has become clear to us that the development of multimeric peptides can be advanced primarily through small-scale, systematic structural variation and comprehensive data collection, rather than by applying theoretical concepts based on determinism or *ad hoc* computational approaches that artificially account for only selected molecular interactions.^7^ Only a solid body of experimental data will ultimately provide the desired improved understanding of the complexity of multivalent probes,^8^ which is needed to promote their implementation in a clinical setting with the goal of improving patient care. This holds especially true since large amounts of real-world data are needed to train deep learning networks, for which complexity and nonlinearity are not intrinsic limitations, and which are currently widely predicted to shape the future of biomedical research.^43^ Finally, this study confirmed that the molecular structure of ^68^Ga-Trivehexin comprises the optimal combination of amino acids within the investigated spectrum of substitution patterns.

## Experimental section

### Materials & methods

Unless otherwise noted, the materials and the instrumentation used (analytical and preparative HPLC, ESI-MS, gamma counter, centrifuge), and the experimental procedures (^68^Ga radiolabeling and radio-TLC for quality control, determination of log *D*_7.4_, cultivation of H2009 human lung adenocarcinoma cells, generation of tumor xenografts in CB17 SCID mice, small-animal PET, and biodistribution) were described before.^19^ All animal experiments have been carried out according to applicable law and institutional guidelines of Technical University of Munich, and were approved by the responsible local authority (Regierung von Oberbayern). The H2009 cells were regularly authenticated and tested for mycoplasma contamination. The affinities of the pentynoic amide functionalized peptides were determined employing a well-established ELISA protocol on immobilized integrins.^36^ TRAP-triazide,^38^FF (previously termed AvB6),^32^ and YY (also referred to as Tyr2-alkyne)^33^ were synthesized as described previously. RP-HPLC for assessment of compound polarity was done on a 150 × 4.6 mm ReproSil-Pur 120 C18-AQ 5 μm column, with a flow of 0.75 mL min^−1^ and a gradient of 10–50% MeCN in water, both containing 0.1% trifluoroacetic acid, within 15 min. The required ^nat^Ga-complexes of the peptide trimers were synthesized by reaction with equimolar amounts of aq. GaCl_3_ as described.^19^ ESI-MS spectra and HPLC chromatograms for the synthesized compounds are provided in Fig. S1–S23.†

### Syntheses

The novel building blocks YF and FY were synthesized in analogy to the detailed description that was published previously for YY.^33^ First, the linear protected peptides YR(Pbf)GD(*t*Bu)LAFp(*N*Me)K(Dde) and FR(Pbf)GD(*t*Bu)LAYp(*N*Me)K(Dde) were assembled employing a solid-phase peptide synthesis Fmoc strategy, during which the backbone *N*-methylation of the Lys was done on-resin after completing Lys-Fmoc deprotection *via* a Mitsunobu approach. After cleavage of the completed peptide chains from the resin, the cyclization was performed in dilute (1 mM) solution using diphenylphosphorylazide/NaHCO_3_. Removal of the Dde protecting group from the Lys side chain afforded the protected cyclic peptides cyclo[FR(Pbf)GD(*t*Bu)LAYp(*N*Me)K] and cyclo[YR(Pbf)GD(*t*Bu)LAFp(*N*Me)K], respectively.

#### Synthesis of FY

Coupling of 4-pentynoic acid to the FY peptide was performed by dissolving 4-pentynoic acid (15.2 mg, 155 μmol, 1.5 eq.), HATU (47 mg, 124 μmol, 1.2 eq.), HOBt (18.9 mg, 124 μmol, 1.2 eq.) and DIPEA (54 μL, 309 μmol, 3 eq.) in a minimum amount of DMF and allowing the mixture to react for 15 min before the dropwise addition to a solution of cyclo[FR(Pbf)GD(*t*Bu)LAYp(*N*Me)K] (147 mg, 103 μmol, 1 eq.) in DMF. The reaction was allowed to proceed for 1.5 h before removing the solvent under pressure, resulting in an orange oil that was directly treated with 3 mL of a mixture of TFA, DCM, water, and TIPS (85 : 10 : 2.5 : 2.5 by volumes). The progression of the deprotection reaction was monitored by HPLC-MS, typically lasting 1–1.5 h. After complete deprotection was observed, the solution was precipitated directly into diethyl ether at a temperature of −20 °C and kept at −20 °C for 2 h to complete precipitation. The precipitate was centrifuged off for 5 min at 1300 rpm, followed immediately by decanting of the organic phase. The precipitated pellet was washed with diethyl ether, centrifuged again, and dried *in vacuo*. The crude product was purified by RP-HPLC as described,^33^ affording FY as a colourless solid with a yield of 55% (64.7 mg, 56.7 μmol).

#### Synthesis of YF

YF was made like FY, by reacting 4-pentynoic acid (7.6 mg, 78 μmol, 1.5 eq.), HATU (23.6 mg, 62.2 μmol, 1.2 eq.), HOBt (9.5 mg, 62.3 μmol, 1.2 eq.) and DIPEA (27 μL, 155 μmol, 3 eq.) with a solution of cyclo[YR(Pbf)GD(*t*Bu)LAFp(*N*Me)K] (74.0 mg, 51.9 μmol, 1 eq.). YF was obtained as a colourless solid with a yield of 76% (45.0 mg, 39.4 μmol).

#### Synthesis of homo-trimers

The homotrimer synthesis was performed by addition of one of the building blocks FF, YF, FY, or YY (3.3 eq.) to a solution of TRAP(azide)_3_ (1 eq.) and copper(ii) acetate (1.2 eq.) in a minimum amount of a mixture of H_2_O and *t*BuOH (4 : 1 by volumes). Sodium ascorbate (50 eq.), dissolved in a minimum volume of H_2_O, was quickly added to the reaction mixture and the solution mixture was vortexed for 1 min, followed by addition of the reaction vial to a heated water bath at 60 °C without stirring. Upon addition of sodium ascorbate to the reaction mixture a green precipitate slowly formed, which dissolved after 2–5 min, resulting in a transparent green solution. The reaction was allowed to progress for 1 h with monitoring of the reaction progression by HPLC-MS. The Cu species were sequestered from the trimer and the reaction solution by addition of 1,4,7-triazacyclononane-1,4,7-triacetic acid (NOTA) (30 eq.) dissolved in H_2_O with adjustment of pH to 2.2 using 12 M HCl. Upon pH adjustment the color of the reaction mixture changed to transparent blue. The demetallation was allowed to progress for 1 h at 60 °C without stirring. The metal-free trimer was then directly purified by RP-HPLC as described before,^33^ followed by lyophilization.

#### Synthesis of hetero-trimers

The syntheses were carried out as described above, with the difference that equimolar amounts (each 1.7 eq.) of two different building blocks (combinations: FF and FY; FF and YF; FF and YY) were used as substrates in a combinatorial approach. Each of these reactions yielded statistical mixtures of four different trimers of both homo- and heteropeptidic composition, of which the desired hetero-trimers Y1F, Y1Y, Y2F, Y2Y, Y2, and Y4 were isolated by semi-preparative HPLC as described before (Table 2).^33^

## Conflicts of interest

N. G. Q. and J. N. are inventors on patent applications related to αvβ6-integrin binding peptide conjugates and ^68^Ga-Trivehexin. J. N. is co-founder and CSO of TRIMT GmbH (Radeberg, Germany) who has licensed IP from TU Munich. J. N. is furthermore a member of the Scientific Advisory Board of Radiopharm Theranostics LLC (Carlton, Australia) who has licensed IP from TRIMT GmbH.

## Supplementary Material

MD-014-D3MD00365E-s001
