# Supplementary material for: Complexity of αvβ6-integrin targeting RGD peptide trimers: emergence of non-specific binding by synergistic interaction
Source: RSC Med Chem. 2023 Sep 28;14(12):2564–73. doi: 10.1039/d3md00365e (PMC10718521; doi:10.1039/d3md00365e)
Supplement: MD-014-D3MD00365E-s001 [file MD-014-D3MD00365E-s001.pdf]

Electronic Supplementary Material (ESI) for RSC Medicinal Chemistry.
This journal is © The Royal Society of Chemistry 2023

Supporting Information

Complexity of αvβ6-Integrin Targeting RGD Peptide Trimers: Emergence of Non-Specific Binding by Synergistic Interaction

Neil Gerard Quigley, Frauke Richter, Susanne Kossatz, Johannes Notni*

1. *Analytical data for cyclic peptide alkyne building blocks* ____ 2

cyclo[FR(Pbf)GD(tBu)LAYp(NMe)K(pentynoic amide)] (FY) ____ 2

cyclo[YR(Pbf)GD(tBu)LAFp(NMe)K(pentynoic amide)] (YF) ____ 3

2. *ESI-MS data for peptide trimers* ____ 4

3. *HPLC Chromatograms of ^{nat}Ga-complexed trimers* ____ 9

4. *Biodistribution data* ____ 14

5. *Kinetic data* ____ 19

1. Analytical data for cyclic peptide alkyne building blocks

*cyclo[FR(Pbf)GD(tBu)LAYp(NMe)K(pentynoic amide)] (**FY**)*

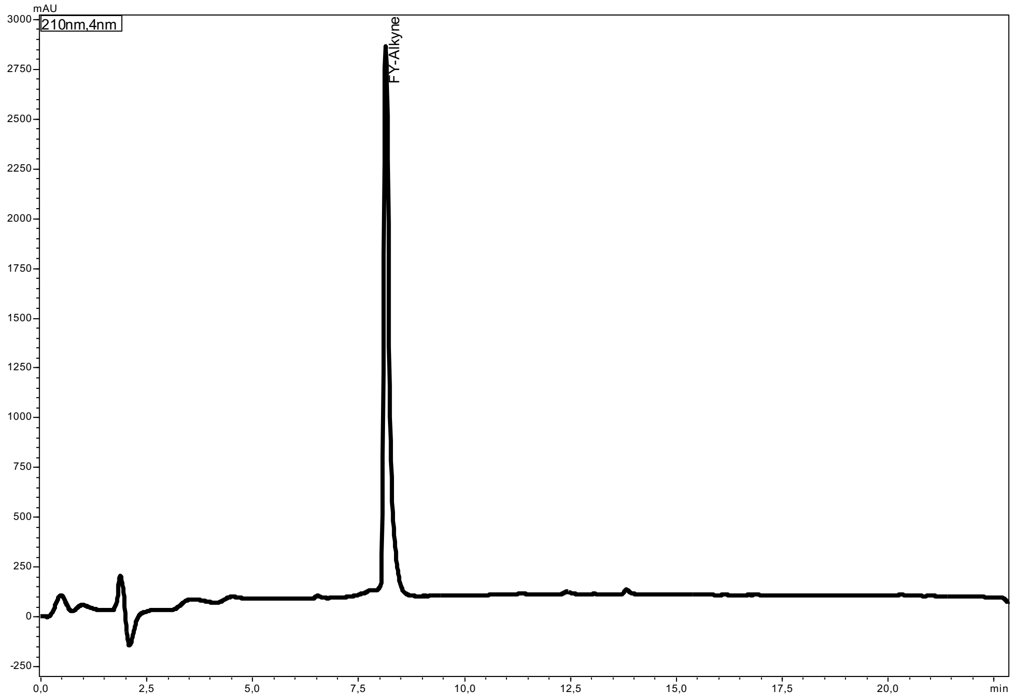

Figure S1: Analytical HPLC (UV 210 nm) of **FY**. HPLC analysis was performed using analytical column C1, 0.5 mL/min; gradient: 10–90% MeCN in H_2O containing 0.1% TFA in 20 min. t_R = 8.2 min.

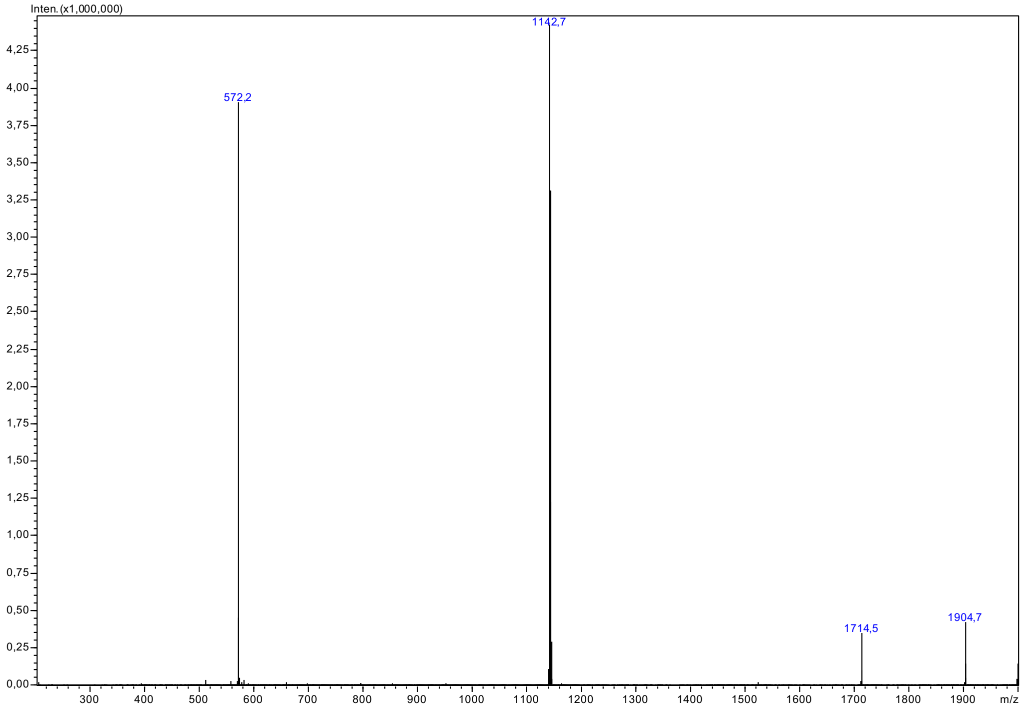

Figure S2: ESI-MS (positive mode) of **FY**. m/z corresponds to molecular composition as follows: 1904.7 $[5M+3H]^{3+}$, 1714.5 $[3M+2H]^{2+}$, 1142.7 $[M+H]^{+}$, 572.2 $[M+2H]^{2+}$.

cyclo[YR(Pbf)GD(tBu)LAFp(NMe)K(pentynoic amide)] (***YF***)

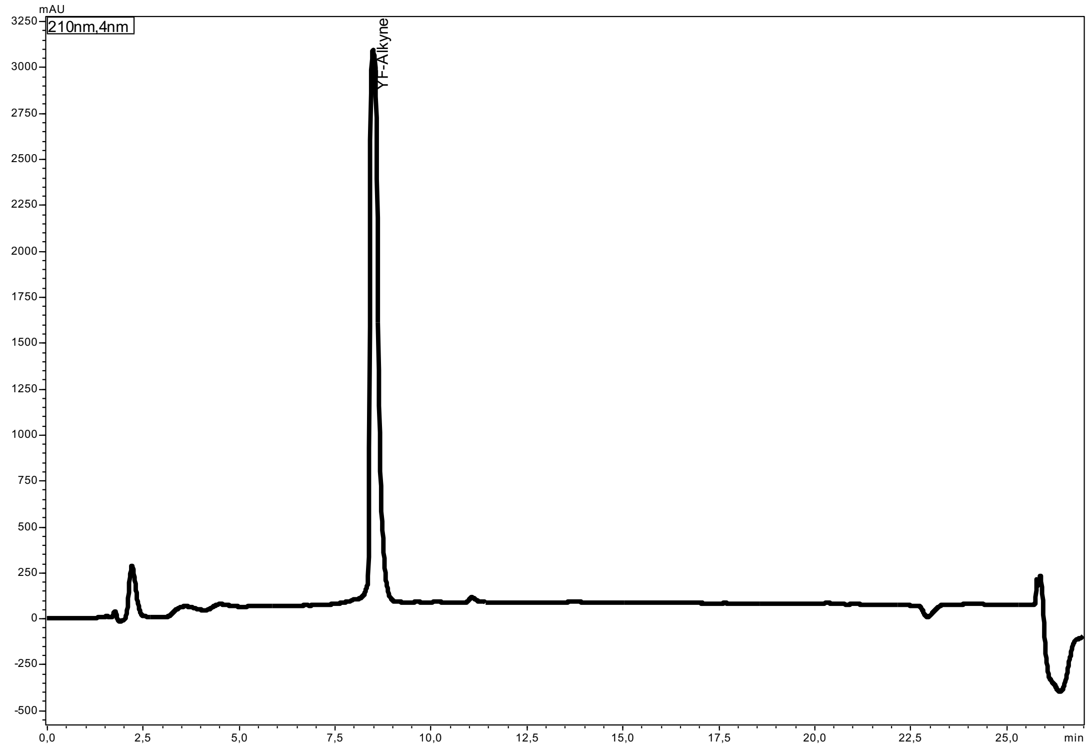

Figure S3: Analytical HPLC (UV 210 nm) of **YF**. HPLC analysis was performed using analytical column C1, 0.5 mL/min; gradient: 10–90% MeCN in H_2O containing 0.1% TFA in 20 min. t_R = 8.2 min.

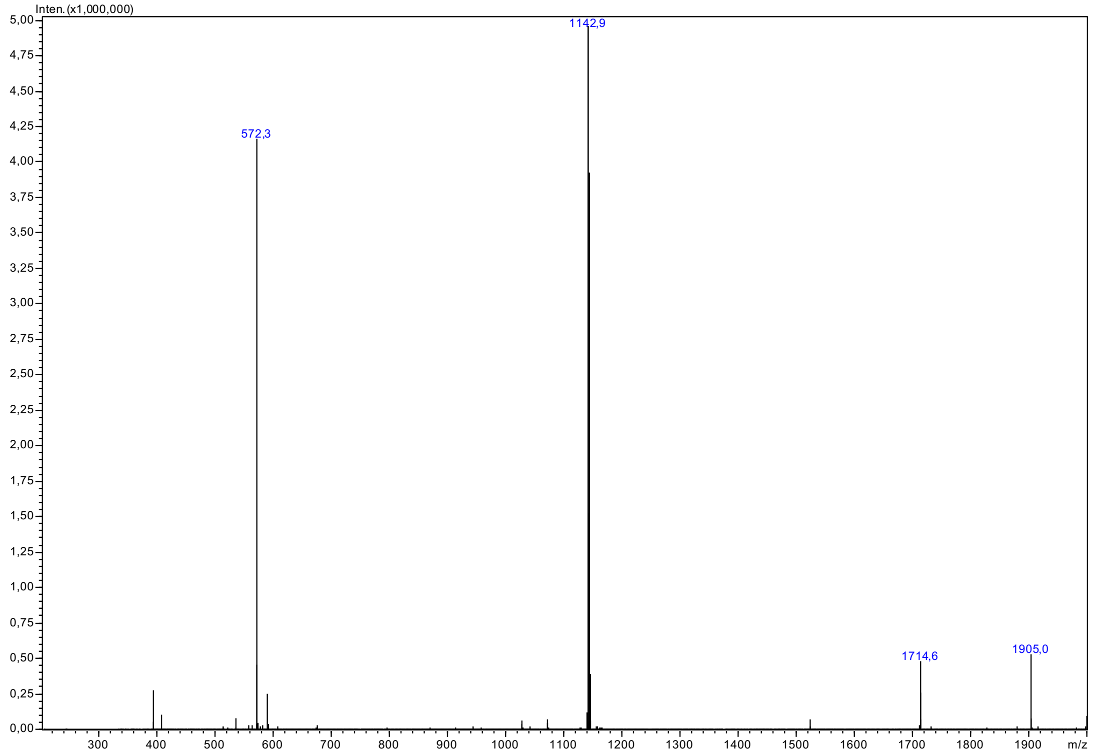

Figure S4: ESI-MS (positive mode) of **YF**. m/z corresponds to molecular composition as follows: 1905 $[5M+3H]^{3+}$, 1714.6 $[3M+2H]^{2+}$, 1142.9 $[M+H]^{+}$, 572.3 $[M+2H]^{2+}$.

2. ESI-MS data for peptide trimers

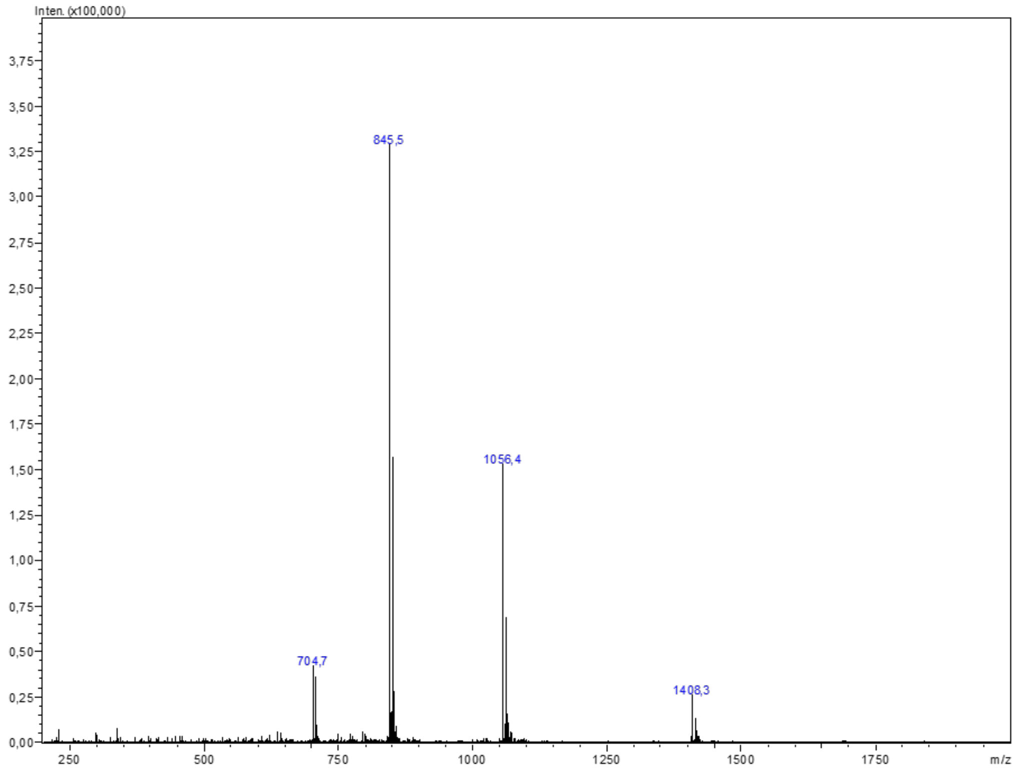

Figure S5: ESI-MS (positive mode) of **Y1Y**. *m/z* corresponds to molecular composition as follows: *m/z* corresponds to molecular composition as follows: 1408.3 $[M+3H]^{3+}$, 1056.4 $[M+4H]^{4+}$, 845.5 $[M+5H]^{5+}$, 704.7 $[M+6H]^{6+}$.

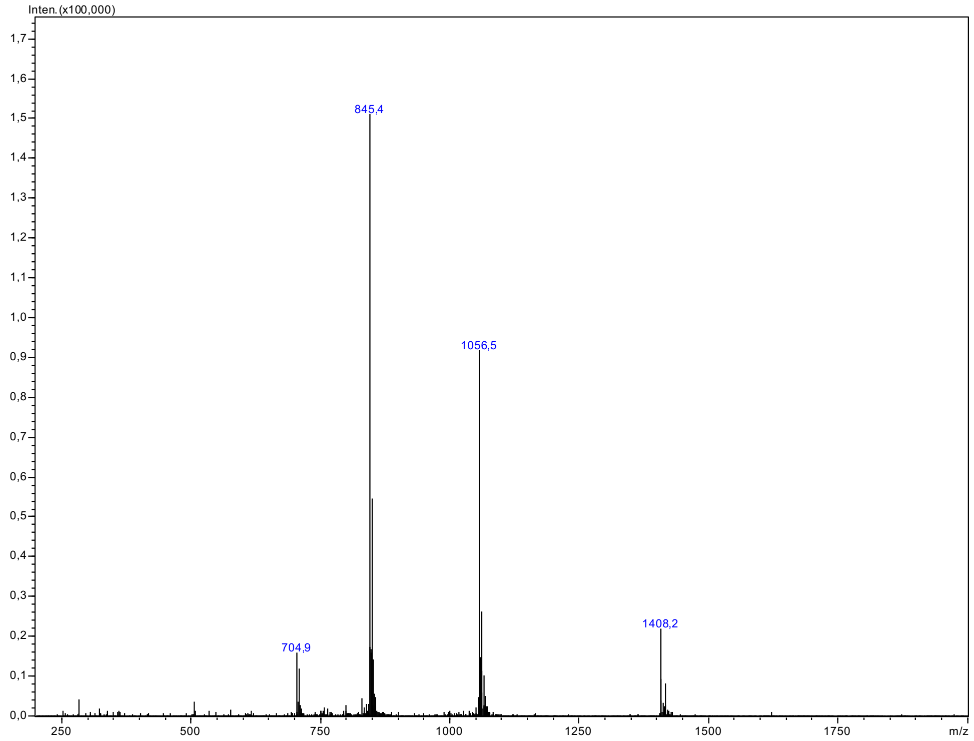

Figure S6: ESI-MS (positive mode) of **Y1F**. *m/z* corresponds to molecular composition as follows: 1408.2 $[M+3H]^{3+}$, 1056.5 $[M+4H]^{4+}$, 845.4 $[M+5H]^{5+}$, 704.9 $[M+6H]^{6+}$.

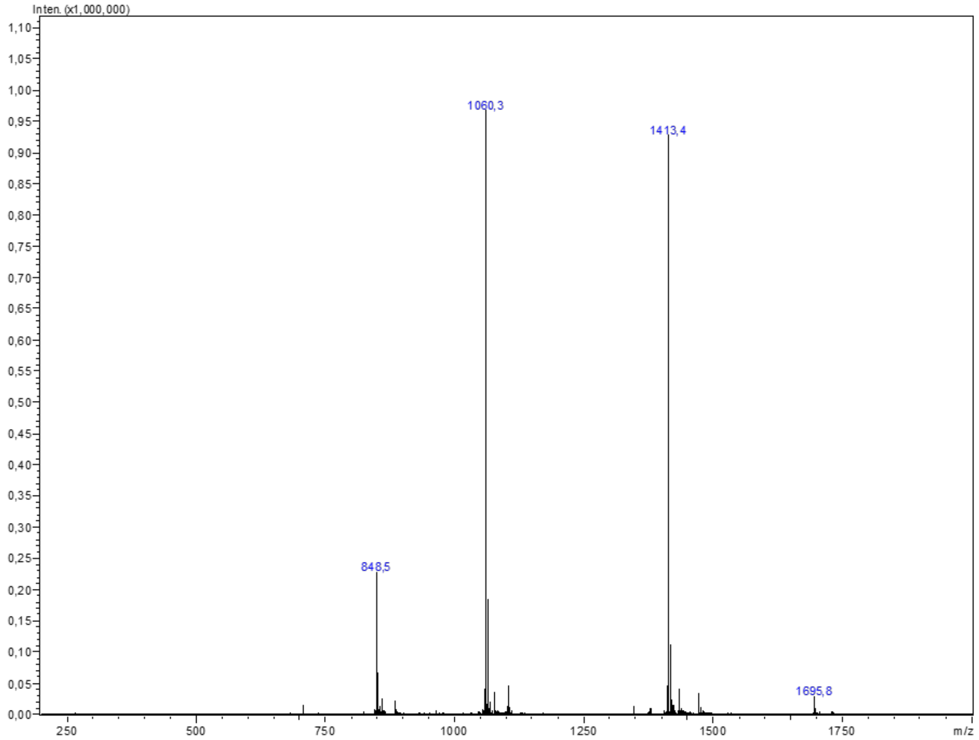

Figure S7: ESI-MS (positive mode) of **Y2.** *m/z* corresponds to molecular composition as follows: *m/z* corresponds to molecular composition as follows: 1695.8 $[2M+5H]^{5+}$, 1413.4 $[M+3H]^{3+}$, 1060.3 $[M+4H]^{4+}$, 848.5 $[M+5H]^{5+}$.

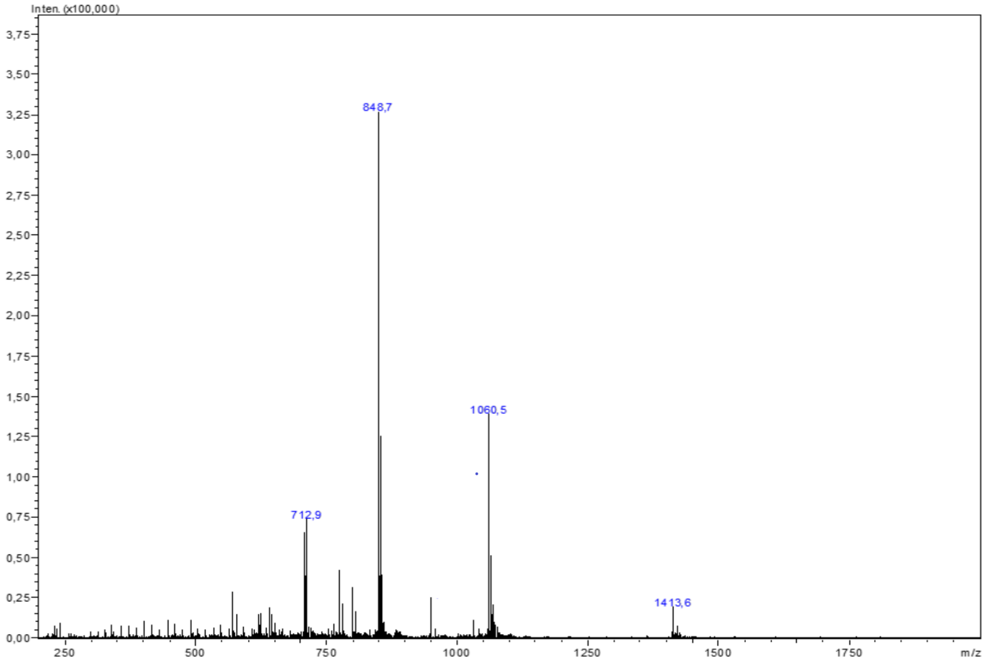

Figure S8: ESI-MS (positive mode) of **Y2Y**. *m/z* corresponds to molecular composition as follows: *m/z* corresponds to molecular composition as follows: 1413.6 $[M+3H]^{3+}$, 1060.5 $[M+4H]^{4+}$, 848.7 $[M+5H]^{5+}$, 712.9 $[M+6H]^{6+}$.

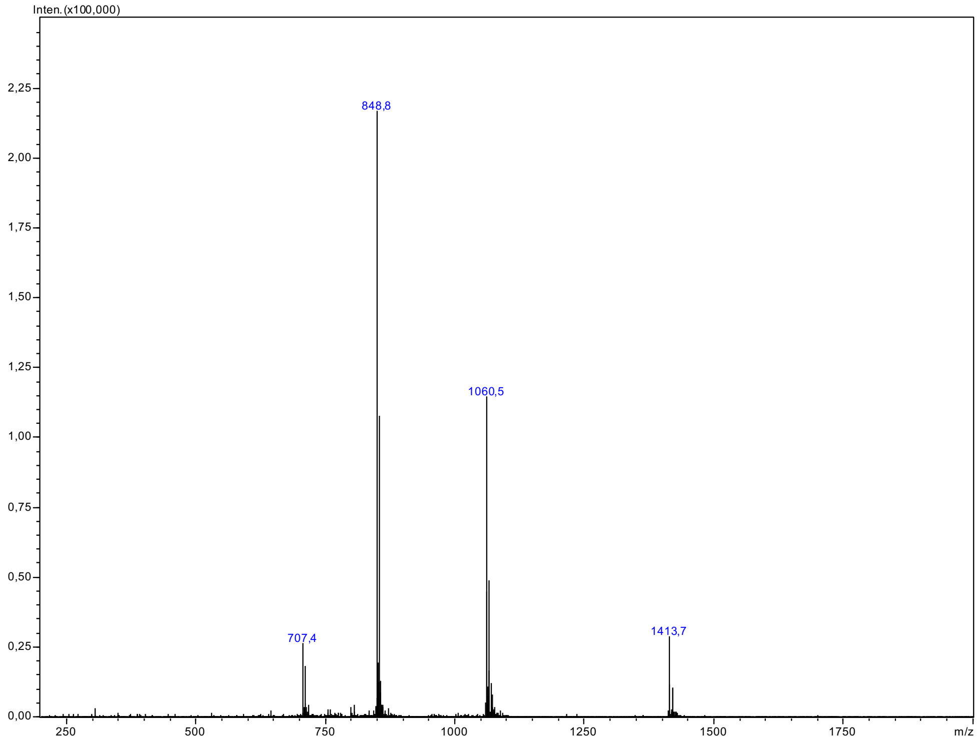

Figure S9: ESI-MS (positive mode) of **Y2F**. *m/z* corresponds to molecular composition as follows: *m/z* corresponds to molecular composition as follows: 1413.7 $[M+3H]^{3+}$, 1060.5 $[M+4H]^{4+}$, 848.8 $[M+5H]^{5+}$, 707.4 $[M+6H]^{6+}$.

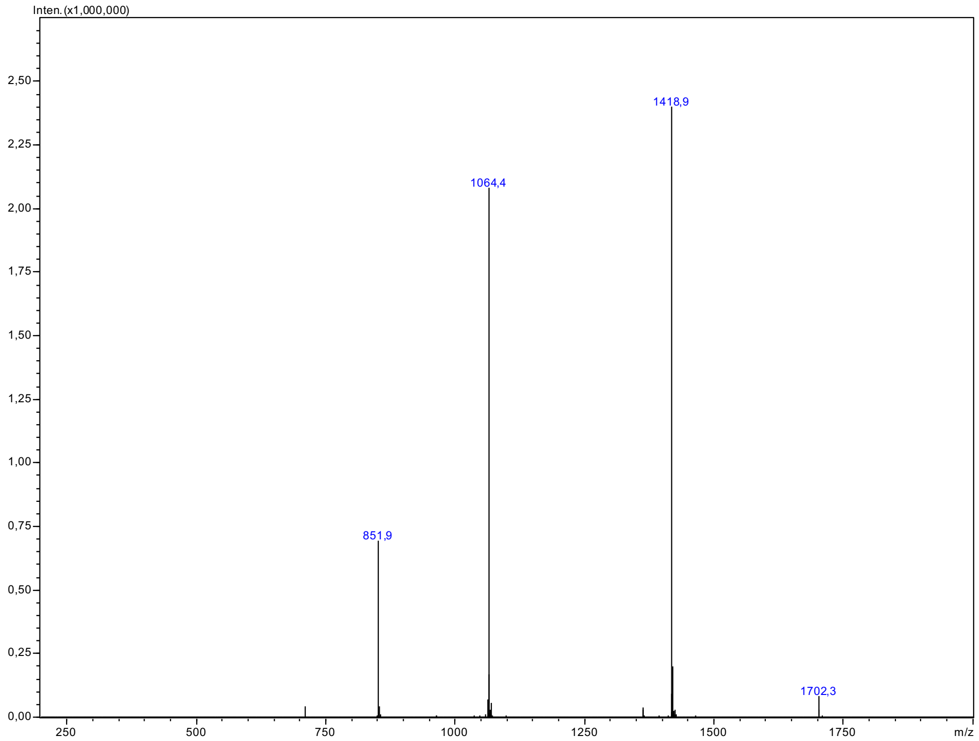

Figure 10: ESI-MS (positive mode) of **Y3Y**. *m/z* corresponds to molecular composition as follows: *m/z* corresponds to molecular composition as follows: 1702.3 $[2M+5H]^{5+}$, 1418.9 $[M+3H]^{3+}$, 1064.4 $[M+4H]^{4+}$, 851.9 $[M+5H]^{5+}$.

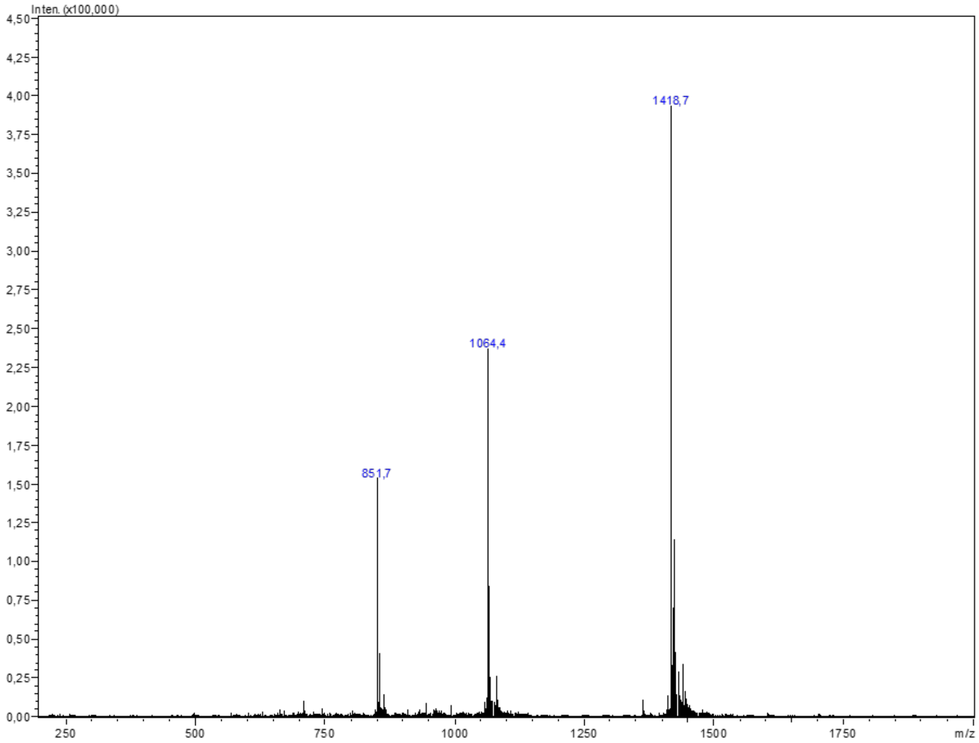

Figure S11: ESI-MS (positive mode) of **Y3F**. *m/z* corresponds to molecular composition as follows: *m/z* corresponds to molecular composition as follows: 1418.7 $[M+3H]^{3+}$, 1064.4 $[M+4H]^{4+}$, 851.7 $[M+5H]^{5+}$.

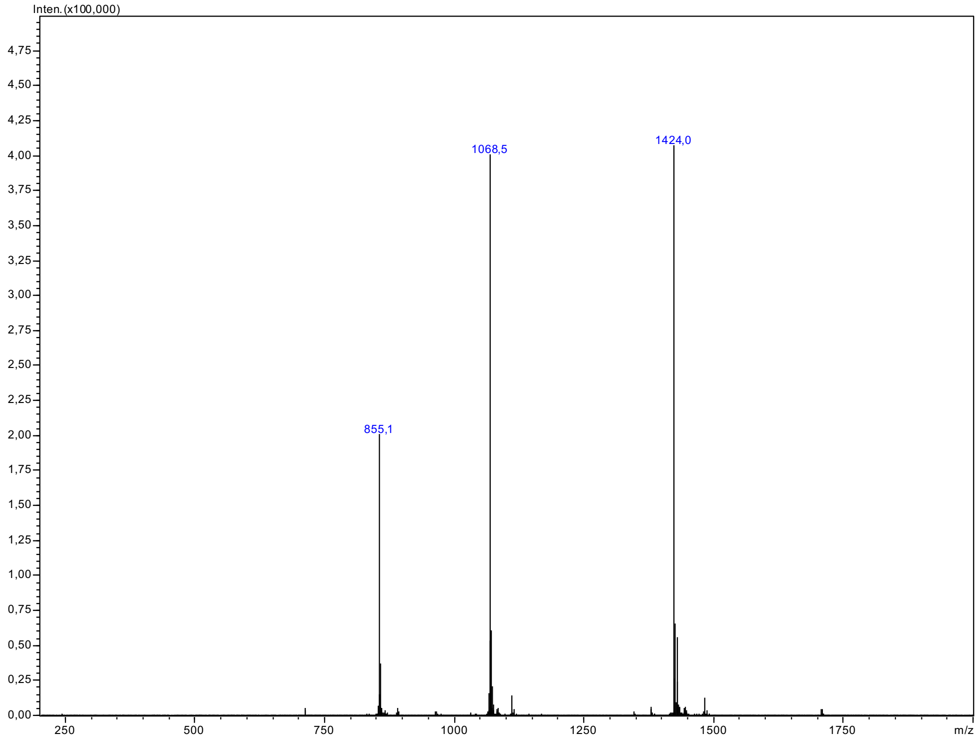

Figure S12: ESI-MS (positive mode) of **Y4**. *m/z* corresponds to molecular composition as follows: *m/z* corresponds to molecular composition as follows: 1424.0 $[M+3H]^{3+}$, 1068.5 $[M+4H]^{4+}$, 855.1 $[M+5H]^{5+}$.

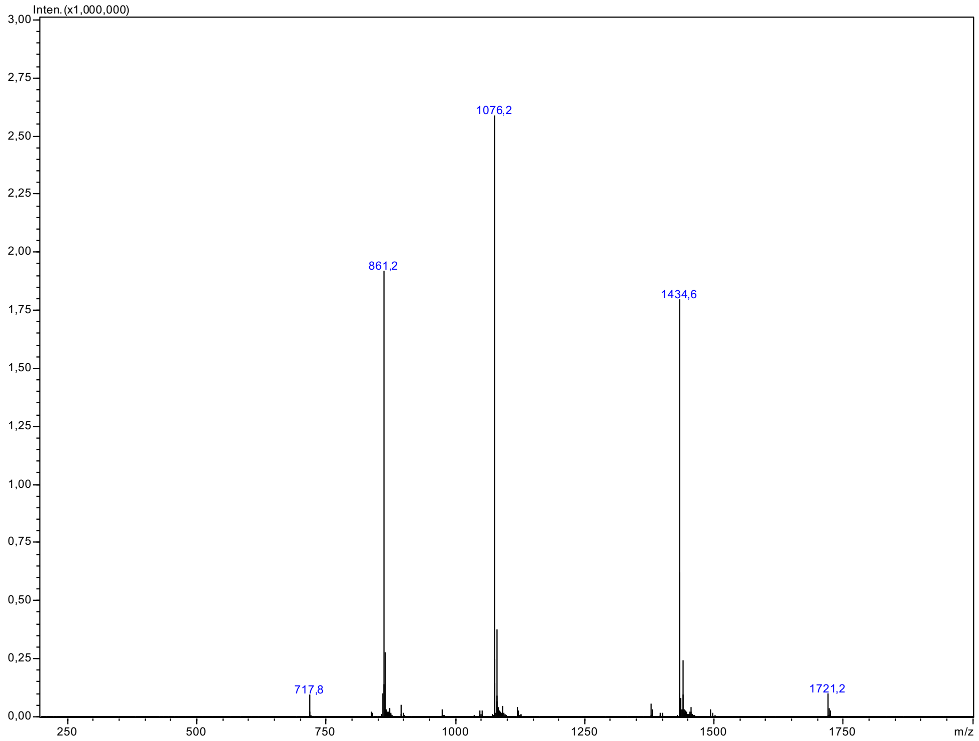

Figure 13: ESI-MS (positive mode) of **Y6**. *m/z* corresponds to molecular composition as follows: *m/z* corresponds to molecular composition as follows: 1721.2 $[2M+5H]^{5+}$, 1434.6 $[M+3H]^{3+}$, 1076.2 $[M+4H]^{4+}$, 861.2 $[M+5H]^{5+}$, 717.8 $[M+6H]^{6+}$.

3. HPLC Chromatograms of ^{nat}Ga-complexed trimers

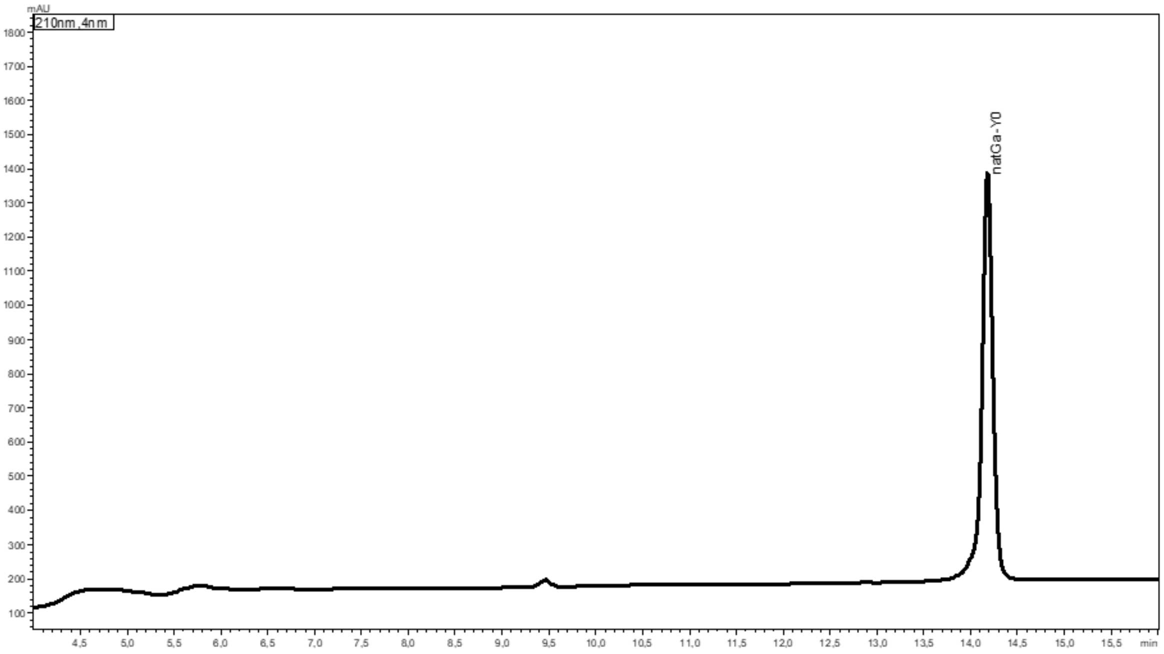

Figure S14: Analytical HPLC (UV 210 nm) of ^{nat}Ga-**Y0**. HPLC analysis was performed using analytical column C2, 0.75 mL/min; gradient: 10–50% MeCN in H_2O containing 0.1% TFA in 15 min. t_R = 14.2 min. Compound Purity = 98% determined by 210 nm wavelength.

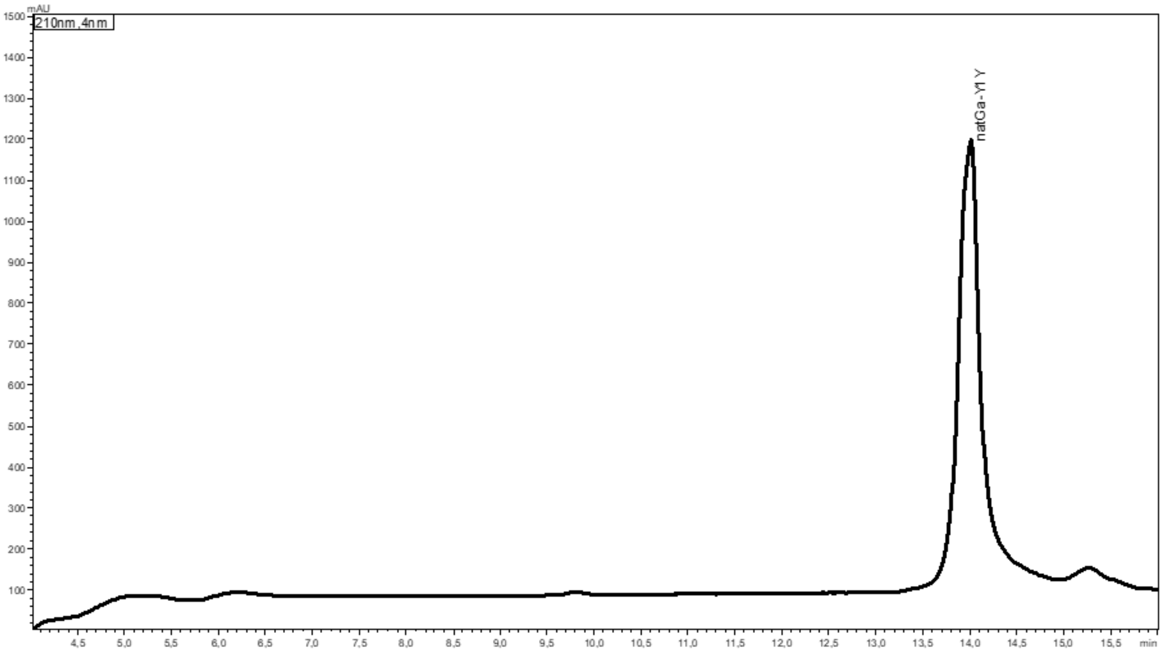

Figure S15: Analytical HPLC (UV 210 nm) of ^{nat}Ga-**Y1Y**. HPLC analysis was performed using analytical column C2, 0.75 mL/min; gradient: 10–50% MeCN in H_2O containing 0.1% TFA in 15 min. t_R = 14.0 min. Compound Purity = 96% determined by 210 nm wavelength.

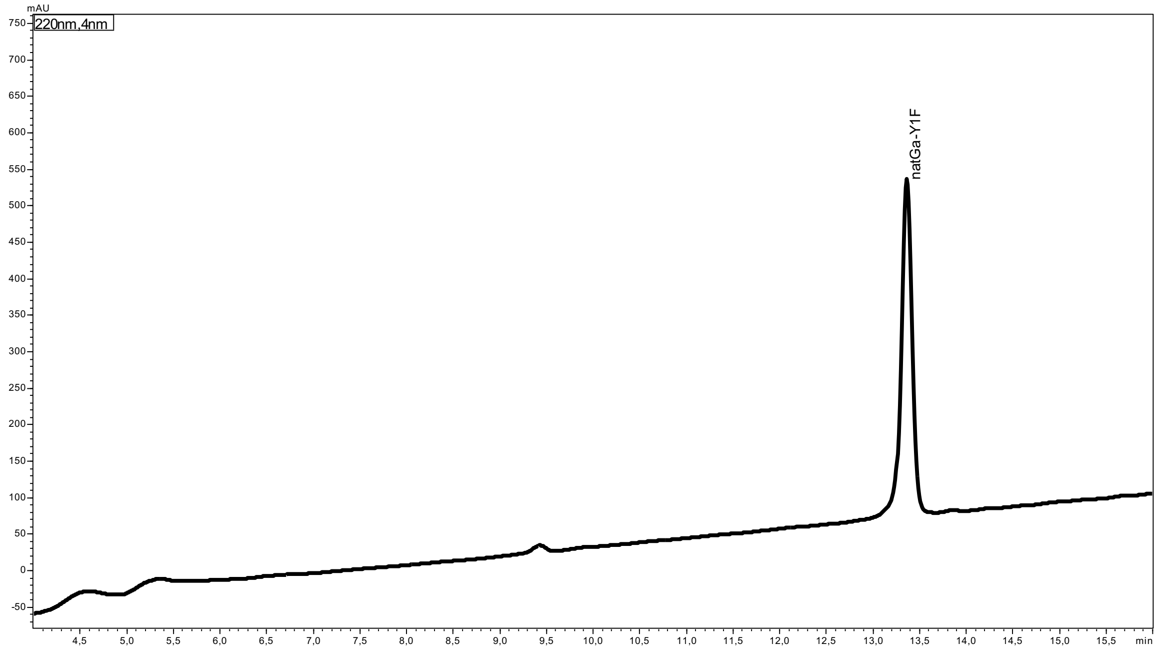

Figure S16: Analytical HPLC-UV (210 nm) of ^{nat}Ga-**Y1F**. HPLC analysis was performed using analytical column C2, 0.75 mL/min; gradient: 10–50% MeCN in H_2O containing 0.1% TFA in 15 min. t_R = 13.4 min. Compound Purity = 98% determined by 210 nm wavelength.

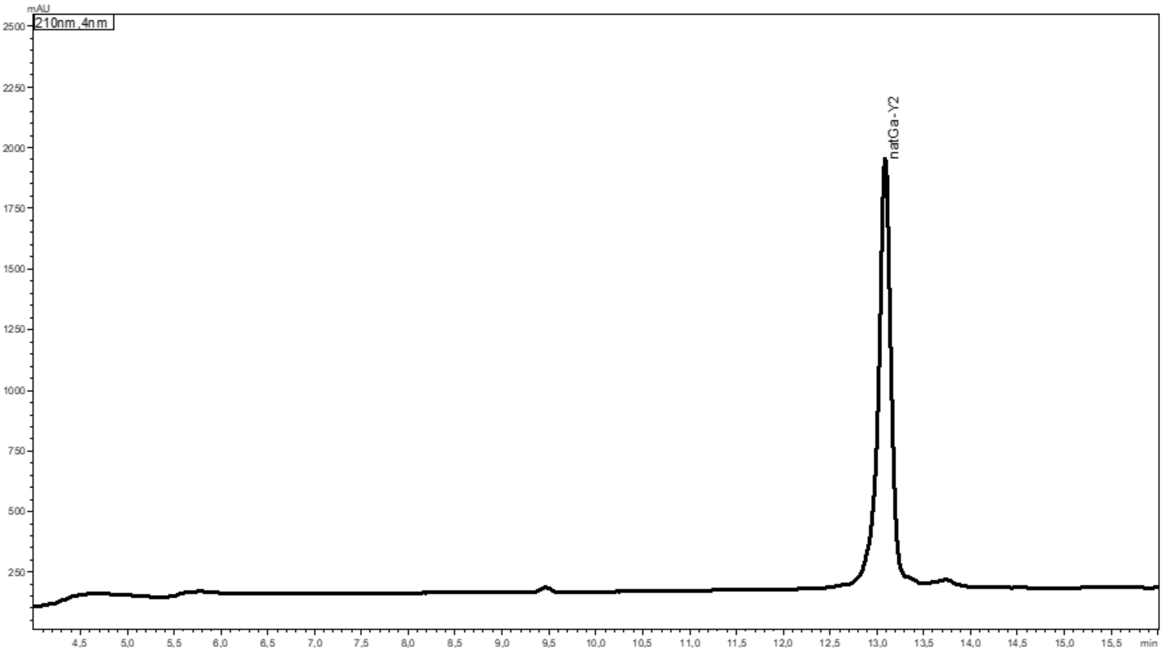

Figure S17: Analytical HPLC (UV 210 nm) of ^{nat}Ga-**Y2**. HPLC analysis was performed using analytical column C2, 0.75 mL/min; gradient: 10–50% MeCN in H_2O containing 0.1% TFA in 15 min. t_R = 13.1 min. Compound Purity = 98% determined by 210 nm wavelength.

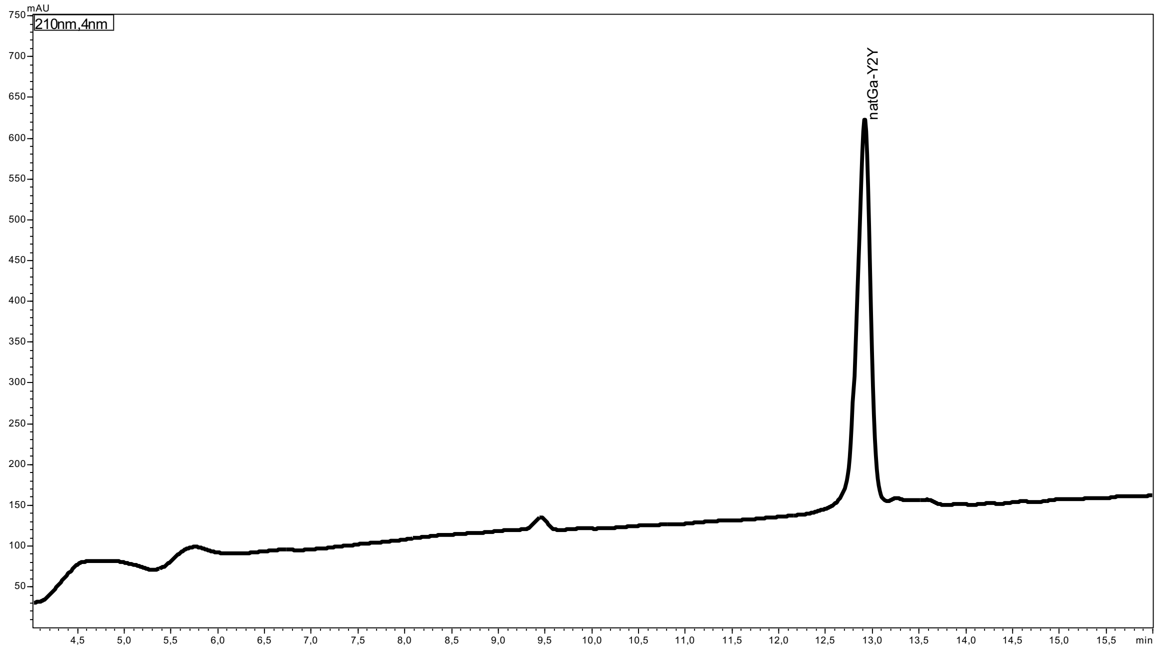

Figure S18: Analytical HPLC (UV 210 nm) of ^{nat}Ga-**Y2Y**. HPLC analysis was performed using analytical column C2, 0.75 mL/min; gradient: 10–50% MeCN in H_2O containing 0.1% TFA in 15 min. t_R = 12.9 min. Compound Purity = 95% determined by 210 nm wavelength.

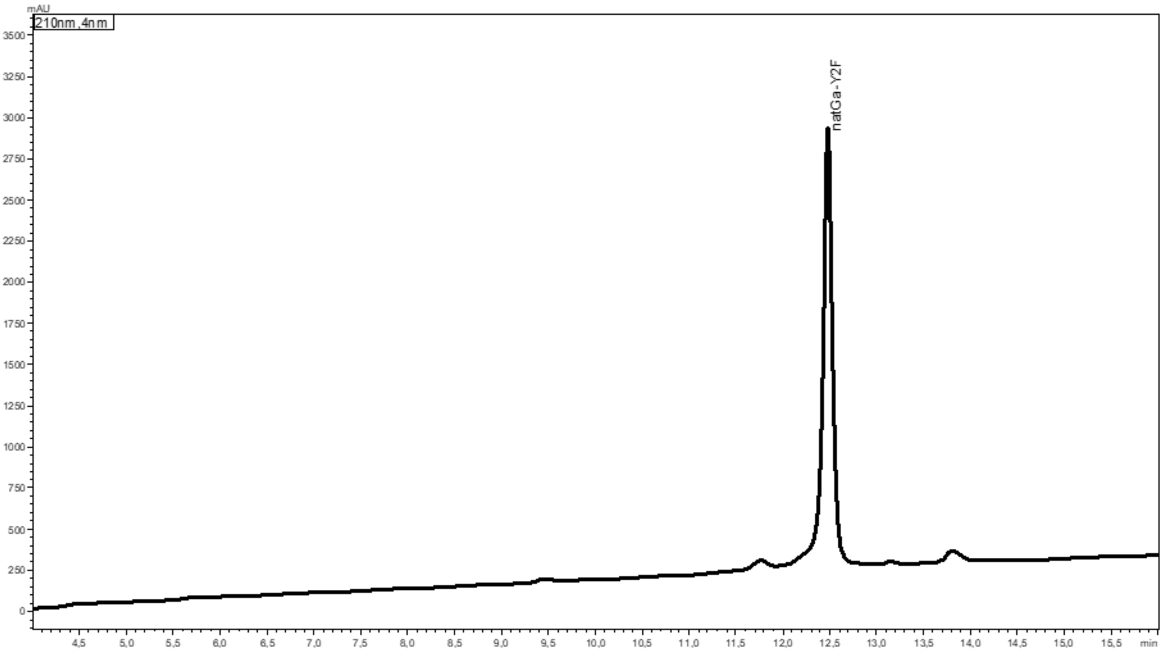

Figure S19: Analytical HPLC (UV 210 nm) of ^{nat}Ga-**Y2F**. HPLC analysis was performed using analytical column C2, 0.75 mL/min; gradient: 10–50% MeCN in H_2O containing 0.1% TFA in 15 min. t_R = 12.5 min. Compound Purity = 95% determined by 210 nm wavelength.

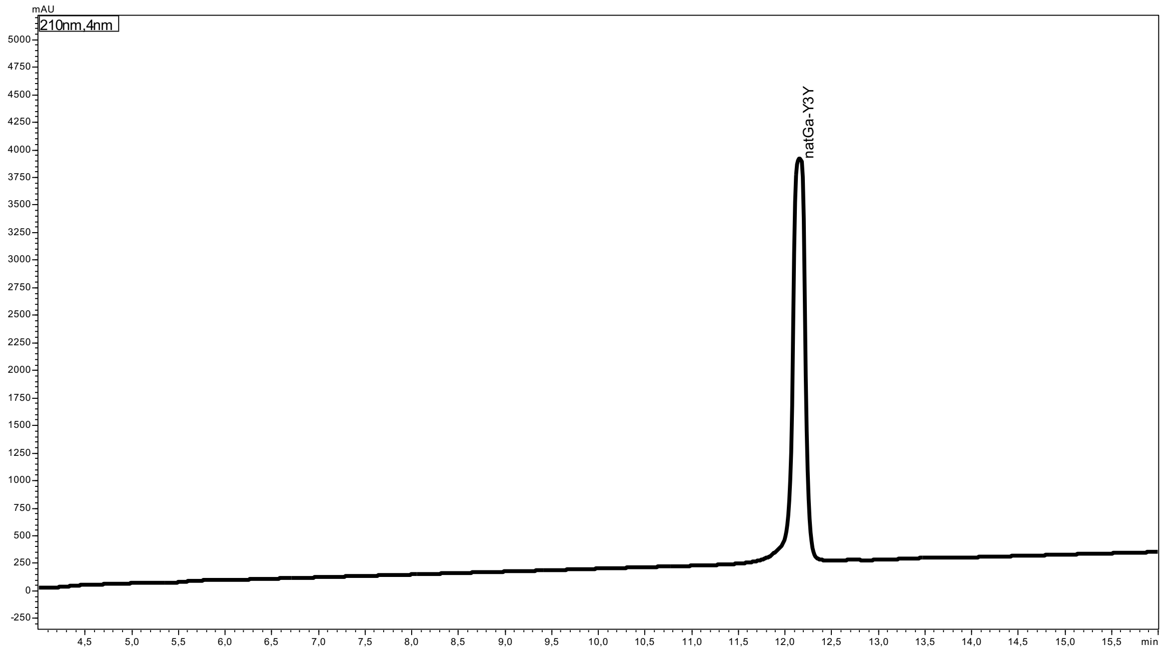

Figure S20: Analytical HPLC (UV 210 nm) of ^{nat}Ga-**Y3Y**. HPLC analysis was performed using analytical column C2, 0.75 mL/min; gradient: 10–50% MeCN in H_2O containing 0.1% TFA in 15 min. t_R = 12.2 min. Compound Purity = 99% determined by 210 nm wavelength.

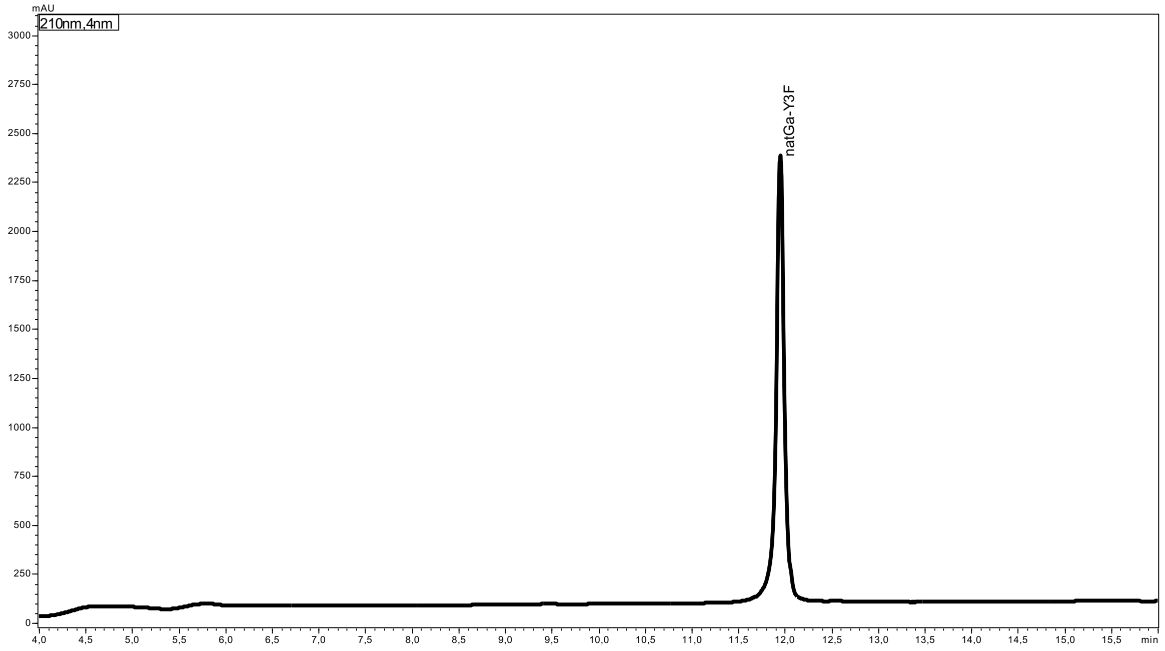

Figure S21: Analytical HPLC (UV 210 nm) of ^{nat}Ga-**Y3F**. HPLC analysis was performed using analytical column C2, 0.75 mL/min; gradient: 10–50% MeCN in H_2O containing 0.1% TFA in 15 min. t_R = 12 min. Compound Purity = 99% determined by 210 nm wavelength.

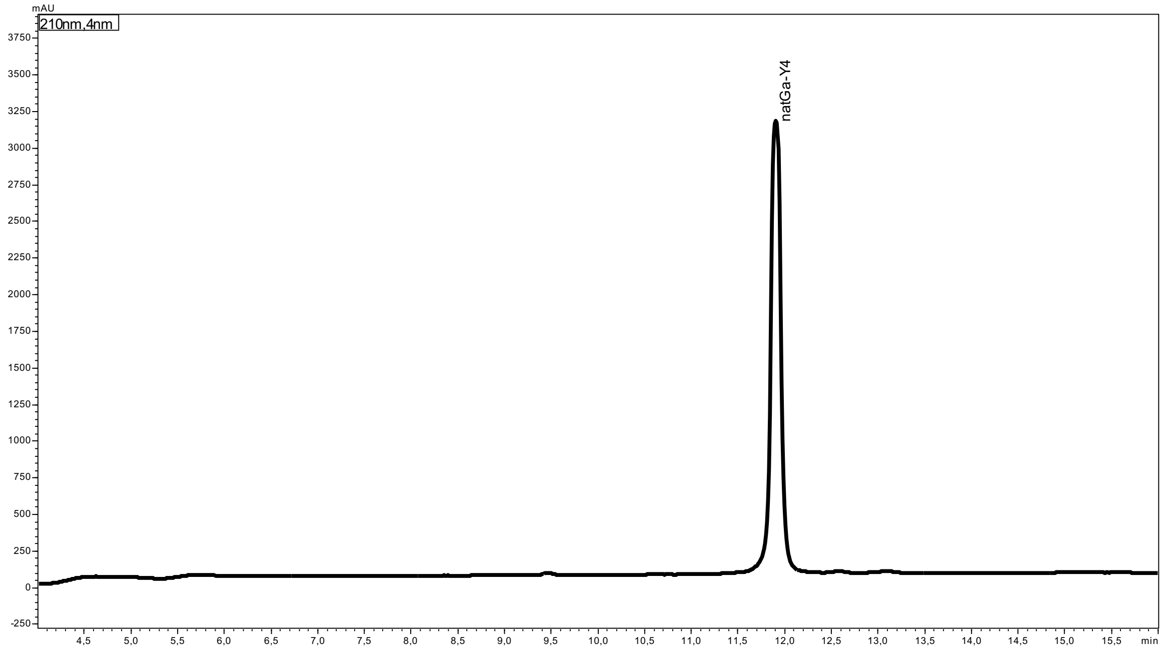

Figure S22: Analytical HPLC (UV 210 nm) of ^{nat}Ga-**Y4**. HPLC analysis was performed using analytical column C2, 0.75 mL/min; gradient: 10–50% MeCN in H_2O containing 0.1% TFA in 15 min. t_R = 11.9 min. Compound Purity = 98% determined by 210 nm wavelength.

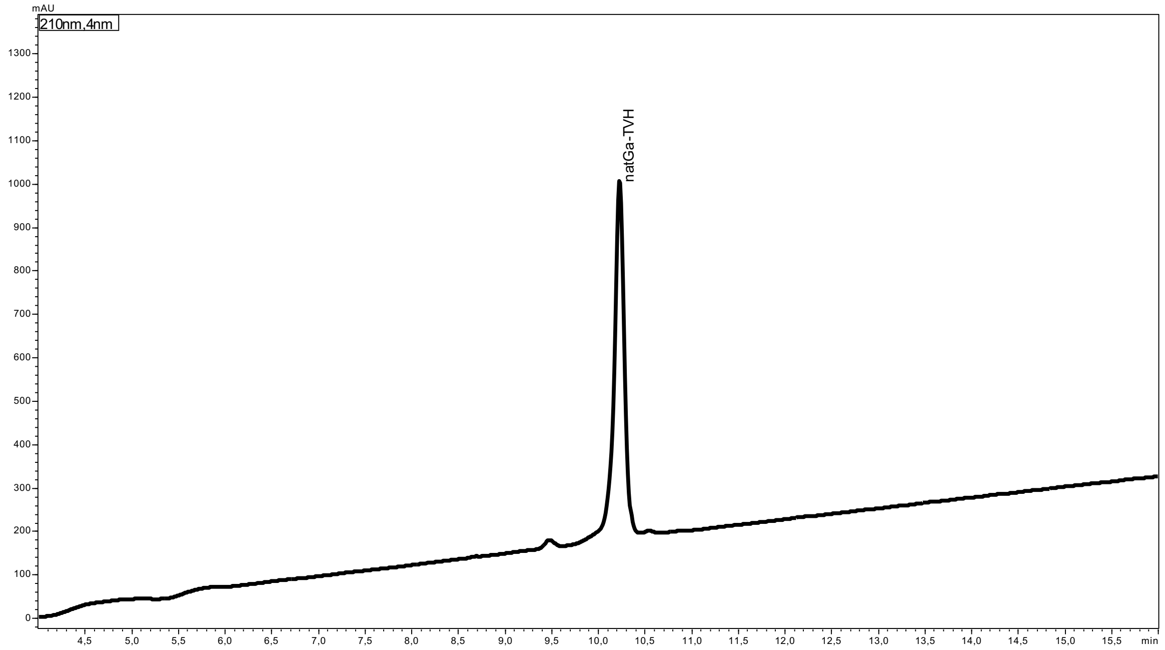

Figure S23: Analytical HPLC (UV 210 nm) of ^{nat}Ga-**Y6** (^{nat}Ga-Trivehexin). HPLC analysis was performed using analytical column C2, 0.75 mL/min; gradient: 10–50% MeCN in H_2O containing 0.1% TFA in 15 min. t_R = 10.2 min. Compound Purity = 98% determined by 210 nm wavelength.

4. Biodistribution data

Table S1: Biodistribution data (90 min p.i.) for ^{68}Ga-**Y0** without (n = 6; 66 ± 8 pmol) and with (n = 3) addition of 50 nmol of TRAP(AvB6)$_3$, in H2009 bearing SCID mice. Data are given as averages ± standard deviation. %IA/g = percent injected activity per gram tissue.

Organ/Tissue	^{68}Ga-**Y0**		+ 50 nmol cold
	%IA/g	tumor/organ ratio	10 min prior to activity
Blood	4.01 ± 0.74	2.2 ± 0.5	2.39 ± 0.79
Heart (myocard)	2.13 ± 0.42	4.2 ± 0.8	1.93 ± 0.37
Lung	16.87 ± 5.34	0.6 ± 0.2	5.66 ± 1.47
Liver	7.55 ± 0.78	1.2 ± 0.2	7.65 ± 1.93
Spleen	5.21 ± 1.05	1.7 ± 0.4	2.56 ± 0.29
Pancreas	0.91 ± 0.22	10.1 ± 2.8	0.99 ± 0.15
Stomach (empty)	10.26 ± 1.62	0.9 ± 0.2	1.46 ± 0.22
Small intestine (empty)	4.20 ± 1.07	2.2 ± 0.4	1.16 ± 0.09
Large intestine (empty)	5.74 ± 1.36	1.6 ± 0.4	1.29 ± 0.16
Kidneys	90.74 ± 6.70	0.1 ± 0.0	61.56 ± 5.90
Adrenals	2.40 ± 0.38	3.7 ± 0.7	1.54 ± 0.43
Muscle	1.10 ± 0.22	8.1 ± 1.3	0.50 ± 0.11
Tumor H2009	8.73 ± 0.81		2.51 ± 0.24

Table S2: Biodistribution data (90 min p.i.) for ^{68}Ga-**Y1Y** without (n = 5; 95 ± 55 pmol) and with (n = 3) addition of 50 nmol of non-labeled compound, in H2009 bearing SCID mice. Data are given as averages ± standard deviation. %IA/g = percent injected activity per gram tissue.

Organ/Tissue	^{68}Ga-**Y1Y**		+ 50 nmol cold
	%IA/g	tumor/organ ratio	10 min prior to activity
Blood	2.54 ± 0.37	3.2 ± 0.7	1.64 ± 0.67
Heart (myocard)	1.54 ± 0.61	5.7 ± 2.0	1.15 ± 0.34
Lung	6.76 ± 2.02	1.2 ± 0.2	3.92 ± 0.97
Liver	4.39 ± 0.46	1.8 ± 0.3	4.61 ± 0.05
Spleen	3.85 ± 0.86	2.2 ± 0.5	2.08 ± 0.25
Pancreas	0.64 ± 0.11	12.7 ± 2.9	0.65 ± 0.12
Stomach (empty)	6.78 ± 0.72	1.2 ± 0.2	1.12 ± 0.19
Small intestine (empty)	2.72 ± 0.36	3.0 ± 0.7	1.11 ± 0.16
Large intestine (empty)	4.10 ± 0.84	2.0 ± 0.5	1.01 ± 0.34
Kidneys	91.21 ± 9.95	0.1 ± 0.0	65.79 ± 13.01
Adrenals	1.57 ± 0.41	5.3 ± 1.5	1.30 ± 0.42
Muscle	0.75 ± 0.17	11.2 ± 3.4	0.34 ± 0.08
Tumor H2009	8.16 ± 2.01		1.50 ± 0.64

Table S3: Biodistribution data (90 min p.i.) for ^{68}Ga-**Y1F** without (n = 5; 68 ± 26 pmol) and with (n = 3) addition of 50 nmol of non-labeled compound, in H2009 bearing SCID mice. Data are given as averages ± standard deviation. %IA/g = percent injected activity per gram tissue.

Organ/Tissue	^{68}Ga-**Y1F**		+ 50 nmol cold
	%IA/g	tumor/organ ratio	10 min prior to activity
Blood	2.57 ± 0.33	3.2 ± 0.6	2.39 ± 0.35
Heart (myocard)	1.20 ± 0.16	6.8 ± 1.2	1.16 ± 0.14
Lung	8.81 ± 0.64	0.9 ± 0.1	4.10 ± 0.93
Liver	3.86 ± 0.17	2.1 ± 0.4	3.30 ± 0.40
Spleen	3.48 ± 0.49	2.4 ± 0.2	1.80 ± 0.47
Pancreas	0.61 ± 0.10	13.3 ± 2.3	0.57 ± 0.11
Stomach (empty)	7.37 ± 1.23	1.1 ± 0.3	1.07 ± 0.14
Small intestine (empty)	2.86 ± 0.57	2.9 ± 0.9	1.07 ± 0.10
Large intestine (empty)	4.43 ± 1.28	2.0 ± 0.9	0.95 ± 0.10
Kidneys	111.14 ± 15.33	0.1 ± 0.0	74.29 ± 4.22
Adrenals	1.26 ± 0.24	6.5 ± 0.8	1.51 ± 0.61
Muscle	0.97 ± 0.48	9.5 ± 3.2	0.36 ± 0.03
Tumor H2009	8.08 ± 1.35		1.80 ± 0.12

Table S4: Biodistribution data (90 min p.i.) for ^{68}Ga-**Y2** without (n = 5; 153 ± 11 pmol) and with (n = 3) addition of 50 nmol of non-labeled compound, in H2009 bearing SCID mice. Data are given as averages ± standard deviation. %IA/g = percent injected activity per gram tissue.

Organ/Tissue	^{68}Ga-**Y2**		+ 50 nmol cold
	%IA/g	tumor/organ ratio	10 min prior to activity
Blood	2.26 ± 0.26	3.5 ± 0.5	1.75 ± 0.34
Heart (myocard)	1.08 ± 0.13	7.4 ± 0.6	0.83 ± 0.03
Lung	4.80 ± 0.50	1.7 ± 0.2	3.15 ± 0.32
Liver	3.37 ± 0.68	2.4 ± 0.4	3.43 ± 0.59
Spleen	2.51 ± 0.33	3.2 ± 0.7	1.91 ± 0.39
Pancreas	0.65 ± 0.12	12.7 ± 2.9	0.57 ± 0.12
Stomach (empty)	7.17 ± 0.58	1.1 ± 0.1	1.15 ± 0.12
Small intestine (empty)	2.55 ± 0.27	3.1 ± 0.5	0.78 ± 0.11
Large intestine (empty)	3.81 ± 0.53	2.1 ± 0.2	0.96 ± 0.13
Kidneys	91.99 ± 12.24	0.1 ± 0.0	71.99 ± 7.05
Adrenals	1.50 ± 1.28	8.8 ± 6.2	1.46 ± 0.35
Muscle	0.72 ± 0.15	11.4 ± 2.4	0.24 ± 0.05
Tumor H2009	7.95 ± 1.02		1.47 ± 0.19

Table S5: Biodistribution data (90 min p.i.) for ^{68}Ga-**Y2Y** without (n = 5; 101 ± 35 pmol) and with (n = 3) addition of 50 nmol of non-labeled compound, in H2009 bearing SCID mice. Data are given as averages ± standard deviation. %IA/g = percent injected activity per gram tissue.

Organ/Tissue	^{68}Ga-**Y2Y**		+ 50 nmol cold
	%IA/g	tumor/organ ratio	10 min prior to activity
Blood	1.91 ± 0.49	4.4 ± 1.1	1.21 ± 0.13
Heart (myocard)	0.85 ± 0.16	9.6 ± 1.8	0.74 ± 0.10
Lung	7.02 ± 1.34	1.2 ± 0.2	3.15 ± 0.69
Liver	2.59 ± 0.35	3.1 ± 0.3	2.37 ± 0.34
Spleen	4.36 ± 0.79	1.9 ± 0.4	1.55 ± 0.15
Pancreas	0.44 ± 0.07	18.2 ± 2.3	0.45 ± 0.10
Stomach (empty)	6.55 ± 1.48	1.3 ± 0.3	0.83 ± 0.18
Small intestine (empty)	2.23 ± 0.41	3.6 ± 0.7	0.52 ± 0.05
Large intestine (empty)	3.89 ± 0.73	2.1 ± 0.4	0.65 ± 0.17
Kidneys	80.77 ± 5.12	0.1 ± 0.0	57.09 ± 6.23
Adrenals	1.07 ± 0.27	7.8 ± 1.7	1.11 ± 0.34
Muscle	0.63 ± 0.12	13.0 ± 2.5	0.27 ± 0.14
Tumor H2009	7.93 ± 0.28		1.21 ± 0.17

Table S6: Biodistribution data (90 min p.i.) for ^{68}Ga-**Y2F** without (n = 5; 110 ± 24 pmol) and with (n = 3) addition of 50 nmol of non-labeled compound, in H2009 bearing SCID mice. Data are given as averages ± standard deviation. %IA/g = percent injected activity per gram tissue.

Organ/Tissue	^{68}Ga-**Y2F**		+ 50 nmol cold
	%IA/g	tumor/organ ratio	10 min prior to activity
Blood	1.62 ± 0.20	4.9 ± 1.0	0.82 ± 0.08
Heart (myocard)	0.76 ± 0.06	10.6 ± 2.0	0.46 ± 0.04
Lung	5.62 ± 1.34	1.4 ± 0.2	2.03 ± 0.09
Liver	2.09 ± 0.17	3.8 ± 0.9	1.86 ± 0.25
Spleen	3.84 ± 1.14	2.2 ± 0.5	1.02 ± 0.38
Pancreas	0.38 ± 0.04	20.8 ± 3.9	0.26 ± 0.06
Stomach (empty)	6.99 ± 3.40	1.3 ± 0.3	0.65 ± 0.13
Small intestine (empty)	2.51 ± 0.50	3.2 ± 0.4	0.55 ± 0.05
Large intestine (empty)	3.96 ± 1.16	2.1 ± 0.5	0.54 ± 0.08
Kidneys	107.37 ± 7.49	0.1 ± 0.0	75.63 ± 7.06
Adrenals	0.84 ± 0.07	9.6 ± 2.2	0.64 ± 0.12
Muscle	0.66 ± 0.12	12.2 ± 1.5	0.14 ± 0.02
Tumor H2009	7.99 ± 1.62		1.03 ± 0.35

Table S7: Biodistribution data (90 min p.i.) for ^{68}Ga-**Y3Y** without (n = 5; 109 ± 7 pmol) and with (n = 3) addition of 50 nmol of non-labeled compound, in H2009 bearing SCID mice. Data are given as averages ± standard deviation. %IA/g = percent injected activity per gram tissue.

Organ/Tissue	^{68}Ga-**Y3Y**		+ 50 nmol cold
	%IA/g	tumor/organ ratio	10 min prior to activity
Blood	1.13 ± 0.25	4.9 ± 0.5	0.56 ± 0.18
Heart (myocard)	0.66 ± 0.16	8.5 ± 1.1	0.45 ± 0.12
Lung	4.96 ± 0.48	1.0 ± 0.2	2.24 ± 0.57
Liver	1.23 ± 0.19	4.4 ± 0.7	1.40 ± 0.43
Spleen	3.82 ± 0.82	1.4 ± 0.2	1.06 ± 0.12
Pancreas	0.33 ± 0.07	17.0 ± 1.4	0.20 ± 0.07
Stomach (empty)	4.03 ± 1.36	1.5 ± 0.7	0.47 ± 0.12
Small intestine (empty)	1.69 ± 0.20	3.2 ± 0.6	0.41 ± 0.15
Large intestine (empty)	3.94 ± 1.85	1.8 ± 1.1	0.58 ± 0.24
Kidneys	72.57 ± 5.25	0.1 ± 0.0	57.16 ± 3.56
Adrenals	0.81 ± 0.41	6.7 ± 3.0	0.29 ± 0.15
Muscle	0.51 ± 0.11	10.7 ± 1.1	0.14 ± 0.08
Tumor H2009	5.92 ± 2.07		0.95 ± 0.14

Table S8: Biodistribution data (90 min p.i.) for ^{68}Ga-**Y3F** without (n = 5; 93 ± 6 pmol) and with (n = 3) addition of 50 nmol of non-labeled compound, in H2009 bearing SCID mice. Data are given as averages ± standard deviation. %IA/g = percent injected activity per gram tissue.

Organ/Tissue	^{68}Ga-**Y3F**		+ 50 nmol cold
	%IA/g	tumor/organ ratio	10 min prior to activity
Blood	0.96 ± 0.30	7.2 ± 1.1	0.43 ± 0.16
Heart (myocard)	0.62 ± 0.15	10.9 ± 0.8	0.34 ± 0.09
Lung	4.83 ± 1.54	1.5 ± 0.3	1.60 ± 0.60
Liver	0.95 ± 0.22	7.1 ± 0.7	1.00 ± 0.40
Spleen	2.38 ± 0.31	2.8 ± 0.4	1.04 ± 0.22
Pancreas	0.34 ± 0.11	20.4 ± 3.1	0.15 ± 0.06
Stomach (empty)	5.64 ± 1.94	1.3 ± 0.3	0.55 ± 0.08
Small intestine (empty)	2.60 ± 0.90	2.7 ± 0.5	0.33 ± 0.07
Large intestine (empty)	5.15 ± 2.63	1.6 ± 0.7	0.42 ± 0.09
Kidneys	71.11 ± 15.01	0.1 ± 0.0	49.29 ± 4.36
Adrenals	0.77 ± 0.42	10.3 ± 3.8	0.75 ± 0.46
Muscle	0.66 ± 0.25	10.9 ± 3.0	0.12 ± 0.07
Tumor H2009	6.69 ± 1.21		0.89 ± 0.29

Table S9: Biodistribution data (90 min p.i.) for ^{68}Ga-**Y4** without (n = 5; 94 ± 14 pmol) and with (n = 3) addition of 50 nmol of non-labeled compound, in H2009 bearing SCID mice. Data are given as averages ± standard deviation. %IA/g = percent injected activity per gram tissue.

Organ/Tissue	^{68}Ga-**Y4**		+ 50 nmol cold
	%IA/g	tumor/organ ratio	10 min prior to activity
Blood	0.86 ± 0.26	5.5 ± 1.4	0.36 ± 0.02
Heart (myocard)	0.47 ± 0.12	9.6 ± 1.3	0.19 ± 0.04
Lung	3.22 ± 0.63	1.4 ± 0.2	1.31 ± 0.36
Liver	0.93 ± 0.14	5.0 ± 0.5	0.84 ± 0.08
Spleen	1.57 ± 0.57	3.0 ± 0.7	1.00 ± 0.17
Pancreas	0.28 ± 0.08	16.5 ± 1.6	0.12 ± 0.00
Stomach (empty)	4.92 ± 1.06	1.0 ± 0.3	0.47 ± 0.03
Small intestine (empty)	1.35 ± 0.53	3.7 ± 1.0	0.27 ± 0.05
Large intestine (empty)	2.29 ± 0.59	2.1 ± 0.6	0.34 ± 0.01
Kidneys	91.29 ± 8.28	0.1 ± 0.0	65.31 ± 8.22
Adrenals	0.71 ± 0.37	7.1 ± 1.9	0.32 ± 0.01
Muscle	01.41 ± 0.06	12.2 ± 2.8	0.07 ± 0.01
Tumor H2009	4.47 ± 0.96		0.82 ± 0.09

Table S10: Biodistribution data (90 min p.i.) for ^{68}Ga-**Y6** (^{68}Ga-Trivehexin) without (n = 5; 97 ± 13 pmol) and with (n = 3) addition of 50 nmol of Trivehexin, in H2009 bearing SCID mice. Data are given as averages ± standard deviation. %IA/g = percent injected activity per gram tissue.

Organ/Tissue	^{68}Ga-**Y6**		+ 50 nmol cold
	%IA/g	tumor/organ ratio	10 min prior to activity
Blood	1.03 ± 0.21	7.5 ± 1.7	0.25 ± 0.15
Heart (myocard)	0.59 ± 0.11	13.2 ± 3.1	0.27 ± 0.03
Lung	4.47 ± 1.12	1.8 ± 0.6	0.98 ± 0.25
Liver	0.73 ± 0.11	10.5 ± 2.2	0.59 ± 0.08
Spleen	3.91 ± 0.91	2.0 ± 0.5	1.14 ± 0.13
Pancreas	0.27 ± 0.04	28.5 ± 4.2	0.10 ± 0.03
Stomach (empty)	6.24 ± 1.24	1.2 ± 0.2	0.37 ± 0.09
Small intestine (empty)	2.72 ± 1.15	3.1 ± 1.1	0.25 ± 0.06
Large intestine (empty)	4.36 ± 0.77	1.8 ± 0.5	0.35 ± 0.08
Kidneys	95.73 ± 12.59	0.1 ± 0.0	80.45 ± 17.63
Adrenals	0.50 ± 0.11	15.9 ± 4.3	0.37 ± 0.13
Muscle	0.68 ± 0.18	11.4 ± 2.6	0.07 ± 0.02
Tumor H2009	7.52 ± 1.21		0.69 ± 0.17

5. Kinetic data

Table S11: Kinetic data for ^{68}Ga-**Y0**, derived from ROI analysis of 90 min dynamic PET scans (n = 3).

Time (min)	Blood	Muscle	Tumor
0.0	0.01 ± 0.00	0.01 ± 0.00	0.01 ± 0.00
0.1	2.15 ± 1.38	0.01 ± 0.00	0.15 ± 0.19
0.2	5.88 ± 1.38	0.01 ± 0.00	0.68 ± 0.17
0.3	9.89 ± 2.02	0.01 ± 0.00	1.26 ± 0.47
0.4	16.32 ± 5.17	0.09 ± 0.07	1.99 ± 0.90
0.5	21.40 ± 8.11	0.51 ± 0.19	2.48 ± 1.32
0.6	23.76 ± 6.69	0.62 ± 0.25	2.75 ± 1.16
0.8	25.90 ± 1.75	0.81 ± 0.08	3.23 ± 1.02
0.9	24.58 ± 0.88	1.01 ± 0.39	3.32 ± 1.08
1.1	23.49 ± 1.06	1.17 ± 0.31	3.44 ± 1.15
1.3	23.00 ± 1.03	1.23 ± 0.24	3.46 ± 1.12
1.4	22.50 ± 1.06	1.36 ± 0.24	3.51 ± 1.19
1.6	22.05 ± 1.16	1.34 ± 0.23	3.59 ± 1.31
1.8	21.75 ± 1.34	1.42 ± 0.15	3.62 ± 1.08
1.9	21.36 ± 1.34	1.25 ± 0.16	3.61 ± 1.09
2.1	21.04 ± 1.42	1.57 ± 0.11	3.53 ± 1.08
2.3	20.81 ± 1.51	1.25 ± 0.06	3.70 ± 1.21
2.4	20.58 ± 1.55	1.36 ± 0.19	3.70 ± 1.22
2.6	20.38 ± 1.59	1.06 ± 0.19	3.74 ± 1.23
2.8	20.08 ± 1.58	1.43 ± 0.08	3.81 ± 1.10
2.9	19.92 ± 1.67	1.13 ± 0.23	3.81 ± 1.20
3.1	19.69 ± 1.44	1.41 ± 0.10	3.86 ± 1.28
3.3	19.56 ± 1.63	1.31 ± 0.26	3.82 ± 1.24
3.4	19.55 ± 1.65	1.37 ± 0.18	3.96 ± 1.27
3.6	19.30 ± 1.81	1.50 ± 0.02	3.96 ± 1.26
3.8	19.17 ± 1.78	1.54 ± 0.12	4.07 ± 1.17
3.9	19.03 ± 1.84	1.18 ± 0.06	4.08 ± 1.36
5.0	18.14 ± 2.04	1.47 ± 0.10	4.29 ± 1.29
7.0	16.72 ± 2.33	1.47 ± 0.11	4.64 ± 1.39
9.0	15.63 ± 2.41	1.55 ± 0.12	4.95 ± 1.53
11.0	14.73 ± 2.45	1.54 ± 0.13	5.21 ± 1.54
13.0	13.97 ± 2.38	1.55 ± 0.16	5.44 ± 1.56
15.0	13.32 ± 2.34	1.65 ± 0.23	5.62 ± 1.62
17.0	12.82 ± 2.24	1.65 ± 0.17	5.79 ± 1.62
19.0	12.39 ± 2.23	1.63 ± 0.18	5.96 ± 1.60
22.5	11.85 ± 2.11	1.68 ± 0.19	6.18 ± 1.62
27.5	11.16 ± 2.00	1.68 ± 0.21	6.45 ± 1.60
32.5	10.64 ± 1.89	1.70 ± 0.25	6.66 ± 1.53
37.5	10.21 ± 1.80	1.69 ± 0.20	6.80 ± 1.52
42.5	9.88 ± 1.75	1.71 ± 0.20	6.92 ± 1.47
47.5	9.63 ± 1.74	1.73 ± 0.19	7.01 ± 1.46
52.5	9.34 ± 1.71	1.71 ± 0.19	7.13 ± 1.36
57.5	9.07 ± 1.68	1.72 ± 0.14	7.20 ± 1.30
65.0	8.72 ± 1.62	1.69 ± 0.17	7.28 ± 1.25
75.0	8.29 ± 1.62	1.68 ± 0.16	7.38 ± 1.10
85.0	7.93 ± 1.58	1.67 0.17	7.45 ± 1.03

Table S12: Kinetic data for ^{68}Ga-**Y1Y**, derived from ROI analysis of 90 min dynamic PET scans (n = 4).

Time (min)	Blood			Muscle			Tumor		
0.0	0.00	±	0.00	0.00	±	0.00	0.00	±	0.00
0.1	2.64	±	2.62	0.00	±	0.00	0.13	±	0.23
0.2	11.28	±	7.21	0.00	±	0.00	0.87	±	0.89
0.3	17.71	±	9.52	0.00	±	0.00	1.66	±	1.34
0.4	24.35	±	12.49	0.00	±	0.00	2.35	±	1.70
0.5	24.10	±	8.21	0.05	±	0.09	2.58	±	1.40
0.6	23.33	±	5.72	0.82	±	0.15	2.54	±	1.20
0.8	24.09	±	3.75	0.91	±	0.28	2.77	±	1.11
0.9	22.88	±	2.77	1.13	±	0.26	2.89	±	1.05
1.1	21.71	±	2.45	1.15	±	0.25	3.03	±	1.00
1.3	23.25	±	2.43	1.21	±	0.14	3.20	±	0.82
1.4	22.69	±	2.49	1.31	±	0.14	3.29	±	0.74
1.6	21.86	±	1.75	1.44	±	0.15	3.37	±	0.76
1.8	21.37	±	1.57	1.39	±	0.26	3.40	±	0.75
1.9	21.10	±	1.57	1.43	±	0.11	3.47	±	0.71
2.1	20.81	±	1.65	1.44	±	0.08	3.58	±	0.76
2.3	20.41	±	1.52	1.46	±	0.12	3.58	±	0.67
2.4	20.39	±	1.72	1.43	±	0.11	3.60	±	0.69
2.6	20.12	±	1.53	1.48	±	0.15	3.67	±	0.65
2.8	19.95	±	1.39	1.40	±	0.17	3.67	±	0.63
2.9	19.66	±	1.57	1.35	±	0.10	3.75	±	0.58
3.1	19.51	±	1.51	1.38	±	0.20	3.81	±	0.59
3.3	19.37	±	1.65	1.51	±	0.09	3.80	±	0.67
3.4	19.05	±	1.62	1.60	±	0.16	3.88	±	0.53
3.6	18.87	±	1.66	1.56	±	0.11	3.82	±	0.61
3.8	18.95	±	1.92	1.54	±	0.05	3.94	±	0.53
3.9	18.56	±	1.47	1.51	±	0.16	3.98	±	0.48
5.0	17.59	±	1.69	1.43	±	0.15	4.17	±	0.43
7.0	15.92	±	1.72	1.47	±	0.14	4.54	±	0.32
9.0	14.60	±	1.72	1.55	±	0.16	4.84	±	0.26
11.0	13.53	±	1.56	1.56	±	0.16	5.04	±	0.23
13.0	12.65	±	1.46	1.59	±	0.15	5.27	±	0.21
15.0	11.88	±	1.51	1.60	±	0.14	5.40	±	0.22
17.0	11.26	±	1.55	1.61	±	0.14	5.56	±	0.23
19.0	10.72	±	1.59	1.60	±	0.11	5.70	±	0.22
22.5	9.90	±	1.50	1.60	±	0.10	5.85	±	0.19
27.5	8.92	±	1.45	1.59	±	0.10	6.05	±	0.19
32.5	8.26	±	1.37	1.60	±	0.08	6.18	±	0.20
37.5	7.76	±	1.28	1.59	±	0.06	6.25	±	0.21
42.5	7.37	±	1.20	1.58	±	0.06	6.33	±	0.24
47.5	7.08	±	1.18	1.54	±	0.05	6.36	±	0.25
52.5	6.80	±	1.11	1.52	±	0.04	6.41	±	0.27
57.5	6.53	±	1.03	1.52	±	0.07	6.43	±	0.30
65.0	6.18	±	0.98	1.47	±	0.06	6.45	±	0.32
75.0	5.74	±	0.87	1.43	±	0.07	6.49	±	0.37
85.0	5.45	±	0.81	1.41		0.09	6.52	±	0.42

Table S13: Kinetic data for ^{68}Ga-**Y1F**, derived from ROI analysis of 90 min dynamic PET scans (n = 2).

Time (min)	Blood			Muscle			Tumor		
0.0	0.99	±	1.41	0.00	±	0.00	0.00	±	0.00
0.1	11.30	±	5.10	0.00	±	0.00	0.35	±	0.49
0.2	18.40	±	4.37	0.03	±	0.05	0.73	±	1.03
0.3	21.84	±	8.22	0.17	±	0.29	0.91	±	1.29
0.4	19.27	±	8.57	0.31	±	0.54	1.19	±	0.86
0.5	17.66	±	7.93	0.34	±	0.59	1.93	±	0.16
0.6	22.31	±	4.99	0.55	±	0.41	2.11	±	0.08
0.8	24.88	±	1.69	0.59	±	0.54	2.27	±	0.02
0.9	23.21	±	0.58	0.73	±	0.63	2.20	±	0.17
1.1	20.72	±	2.42	0.66	±	0.57	2.27	±	0.19
1.3	20.92	±	1.47	0.75	±	0.66	2.29	±	0.18
1.4	20.67	±	1.62	0.82	±	0.48	2.17	±	0.42
1.6	20.11	±	1.34	0.92	±	0.37	2.36	±	0.16
1.8	19.56	±	1.48	1.03	±	0.22	2.46	±	0.36
1.9	19.36	±	1.43	1.18	±	0.16	2.40	±	0.23
2.1	18.91	±	1.27	1.19	±	0.08	2.50	±	0.35
2.3	18.69	±	1.28	1.33	±	0.09	2.49	±	0.13
2.4	18.54	±	1.35	1.23	±	0.10	2.54	±	0.42
2.6	18.29	±	1.43	1.36	±	0.20	2.65	±	0.23
2.8	18.04	±	1.39	1.30	±	0.14	2.52	±	0.37
2.9	17.81	±	0.99	1.31	±	0.13	2.74	±	0.23
3.1	17.74	±	1.28	1.44	±	0.12	2.93	±	0.11
3.3	17.31	±	1.22	1.38	±	0.22	2.82	±	0.18
3.4	17.07	±	1.20	1.37	±	0.22	2.98	±	0.24
3.6	16.89	±	1.20	1.49	±	0.12	2.88	±	0.02
3.8	16.66	±	1.35	1.41	±	0.14	3.08	±	0.14
3.9	16.61	±	1.35	1.47	±	0.29	2.97	±	0.06
5.0	15.58	±	1.52	1.41	±	0.19	3.35	±	0.05
7.0	13.91	±	1.59	1.51	±	0.17	3.74	±	0.08
9.0	12.47	±	1.40	1.49	±	0.19	4.09	±	0.10
11.0	11.25	±	0.99	1.51	±	0.17	4.37	±	0.07
13.0	10.31	±	0.73	1.55	±	0.15	4.61	±	0.02
15.0	9.70	±	0.57	1.58	±	0.19	4.78	±	0.00
17.0	9.24	±	0.42	1.57	±	0.18	4.96	±	0.06
19.0	8.85	±	0.19	1.60	±	0.17	5.09	±	0.03
22.5	8.42	±	0.05	1.59	±	0.21	5.23	±	0.11
27.5	7.87	±	0.24	1.59	±	0.20	5.46	±	0.07
32.5	7.49	±	0.37	1.57	±	0.22	5.62	±	0.09
37.5	7.19	±	0.48	1.58	±	0.20	5.78	±	0.11
42.5	6.95	±	0.48	1.57	±	0.25	5.86	±	0.09
47.5	6.71	±	0.47	1.58	±	0.20	5.97	±	0.14
52.5	6.50	±	0.38	1.55	±	0.24	6.03	±	0.12
57.5	6.38	±	0.38	1.54	±	0.20	6.05	±	0.11
65.0	6.20	±	0.36	1.52	±	0.24	6.16	±	0.07
75.0	5.94	±	0.33	1.51	±	0.24	6.24	±	0.06
85.0	5.71	±	0.31	1.47		0.22	6.35	±	0.05

Table S14: Kinetic data for ^{68}Ga-**Y2**, derived from ROI analysis of 90 min dynamic PET scans (n = 3).

Time (min)	Blood	Muscle	Tumor
0.0	0.75 ± 1.28	0.00 ± 0.00	0.00 ± 0.00
0.1	6.94 ± 7.46	0.00 ± 0.00	0.11 ± 0.09
0.2	19.50 ± 10.00	0.00 ± 0.00	0.89 ± 0.49
0.3	27.64 ± 8.30	0.01 ± 0.01	1.66 ± 0.53
0.4	30.18 ± 1.67	0.26 ± 0.42	2.37 ± 0.50
0.5	28.66 ± 2.23	0.58 ± 0.58	2.83 ± 0.40
0.6	24.65 ± 0.43	0.95 ± 0.20	2.95 ± 0.86
0.8	22.29 ± 1.32	1.21 ± 0.06	3.16 ± 1.06
0.9	21.50 ± 1.21	1.44 ± 0.09	3.41 ± 0.99
1.1	20.61 ± 0.90	1.33 ± 0.06	3.52 ± 0.76
1.3	20.30 ± 1.16	1.46 ± 0.25	3.51 ± 0.79
1.4	19.74 ± 0.67	1.43 ± 0.20	3.60 ± 0.82
1.6	19.02 ± 1.14	1.24 ± 0.14	3.62 ± 0.74
1.8	18.98 ± 0.96	1.46 ± 0.07	3.69 ± 0.85
1.9	18.48 ± 0.84	1.53 ± 0.15	3.82 ± 0.80
2.1	18.41 ± 0.96	1.67 ± 0.25	3.88 ± 0.75
2.3	17.83 ± 0.69	1.58 ± 0.32	3.86 ± 0.82
2.4	17.59 ± 0.71	1.54 ± 0.14	4.08 ± 0.63
2.6	17.29 ± 0.72	1.49 ± 0.19	4.03 ± 0.79
2.8	16.94 ± 0.73	1.72 ± 0.11	4.03 ± 0.70
2.9	16.67 ± 0.49	1.63 ± 0.09	4.10 ± 0.69
3.1	16.46 ± 0.56	1.72 ± 0.12	4.14 ± 0.78
3.3	16.32 ± 0.39	1.72 ± 0.34	4.26 ± 0.67
3.4	15.95 ± 0.32	1.72 ± 0.03	4.24 ± 0.76
3.6	15.88 ± 0.28	1.81 ± 0.16	4.36 ± 0.75
3.8	15.65 ± 0.27	1.69 ± 0.03	4.36 ± 0.66
3.9	15.27 ± 0.16	1.82 ± 0.34	4.53 ± 0.52
5.0	14.17 ± 0.22	1.68 ± 0.13	4.62 ± 0.64
7.0	12.45 ± 0.51	1.71 ± 0.15	4.88 ± 0.57
9.0	11.30 ± 0.68	1.74 ± 0.16	5.09 ± 0.54
11.0	10.48 ± 0.75	1.73 ± 0.20	5.23 ± 0.52
13.0	9.85 ± 0.79	1.71 ± 0.20	5.30 ± 0.53
15.0	9.34 ± 0.81	1.68 ± 0.15	5.35 ± 0.54
17.0	8.95 ± 0.81	1.65 ± 0.14	5.44 ± 0.52
19.0	8.59 ± 0.86	1.65 ± 0.14	5.44 ± 0.58
22.5	8.16 ± 0.84	1.64 ± 0.17	5.46 ± 0.59
27.5	7.70 ± 0.87	1.61 ± 0.16	5.50 ± 0.61
32.5	7.26 ± 0.87	1.57 ± 0.17	5.48 ± 0.65
37.5	6.92 ± 0.84	1.54 ± 0.15	5.48 ± 0.65
42.5	6.62 ± 0.82	1.49 ± 0.17	5.47 ± 0.67
47.5	6.33 ± 0.80	1.49 ± 0.18	5.47 ± 0.70
52.5	6.09 ± 0.81	1.44 ± 0.15	5.43 ± 0.70
57.5	5.83 ± 0.80	1.42 ± 0.14	5.44 ± 0.69
65.0	5.51 ± 0.81	1.38 ± 0.16	5.45 ± 0.69
75.0	5.13 ± 0.86	1.34 ± 0.16	5.41 ± 0.67
85.0	4.80 ± 0.84	1.30 0.17	5.40 ± 0.66

Table S15: Kinetic data for ^{68}Ga-**Y2Y**, derived from ROI analysis of 90 min dynamic PET scans (n = 3).

Time (min)	Blood	Muscle	Tumor
0.0	0.00 ± 0.00	0.00 ± 0.00	0.00 ± 0.00
0.1	3.95 ± 1.48	0.00 ± 0.00	0.12 ± 0.12
0.2	12.14 ± 0.76	0.00 ± 0.00	0.81 ± 0.13
0.3	18.86 ± 0.49	0.11 ± 0.18	1.65 ± 0.26
0.4	24.31 ± 3.17	0.18 ± 0.29	2.40 ± 0.57
0.5	24.43 ± 6.95	0.61 ± 0.34	2.73 ± 0.89
0.6	20.59 ± 4.39	1.09 ± 0.26	2.65 ± 0.84
0.8	19.62 ± 3.96	1.18 ± 0.31	2.90 ± 0.96
0.9	19.12 ± 3.65	1.21 ± 0.23	2.93 ± 0.89
1.1	18.76 ± 3.55	1.27 ± 0.31	3.03 ± 0.90
1.3	18.14 ± 3.49	1.43 ± 0.42	3.11 ± 0.91
1.4	17.95 ± 3.53	1.37 ± 0.41	3.15 ± 0.89
1.6	17.48 ± 3.42	1.39 ± 0.28	3.23 ± 0.92
1.8	17.34 ± 3.31	1.30 ± 0.31	3.33 ± 0.88
1.9	16.86 ± 3.40	1.37 ± 0.33	3.38 ± 0.89
2.1	16.69 ± 3.33	1.46 ± 0.32	3.40 ± 0.94
2.3	16.43 ± 3.26	1.38 ± 0.40	3.45 ± 0.89
2.4	16.34 ± 3.32	1.51 ± 0.26	3.47 ± 0.90
2.6	16.09 ± 3.32	1.43 ± 0.34	3.52 ± 0.87
2.8	15.78 ± 3.08	1.37 ± 0.36	3.60 ± 0.89
2.9	15.67 ± 3.34	1.61 ± 0.41	3.66 ± 0.87
3.1	15.42 ± 3.24	1.39 ± 0.22	3.74 ± 0.90
3.3	15.35 ± 3.23	1.49 ± 0.53	3.77 ± 0.90
3.4	15.11 ± 3.22	1.47 ± 0.28	3.77 ± 0.89
3.6	14.84 ± 3.21	1.40 ± 0.39	3.83 ± 0.94
3.8	14.82 ± 3.35	1.56 ± 0.46	3.87 ± 0.90
3.9	14.64 ± 3.04	1.53 ± 0.33	3.93 ± 0.90
5.0	13.69 ± 3.02	1.52 ± 0.33	4.14 ± 0.91
7.0	12.37 ± 2.81	1.59 ± 0.38	4.52 ± 0.96
9.0	11.31 ± 2.64	1.62 ± 0.41	4.79 ± 1.01
11.0	10.42 ± 2.48	1.63 ± 0.41	5.00 ± 1.04
13.0	9.73 ± 2.44	1.65 ± 0.45	5.19 ± 1.07
15.0	9.10 ± 2.33	1.63 ± 0.43	5.34 ± 1.11
17.0	8.53 ± 2.26	1.65 ± 0.47	5.47 ± 1.19
19.0	8.09 ± 2.17	1.65 ± 0.46	5.56 ± 1.21
22.5	7.47 ± 2.03	1.63 ± 0.46	5.74 ± 1.29
27.5	6.77 ± 1.88	1.62 ± 0.49	5.90 ± 1.36
32.5	6.24 ± 1.75	1.58 ± 0.53	6.01 ± 1.41
37.5	5.82 ± 1.65	1.57 ± 0.52	6.10 ± 1.44
42.5	5.54 ± 1.61	1.55 ± 0.53	6.18 ± 1.50
47.5	5.31 ± 1.60	1.54 ± 0.52	6.23 ± 1.52
52.5	5.10 ± 1.58	1.52 ± 0.51	6.26 ± 1.53
57.5	4.89 ± 1.50	1.47 ± 0.49	6.29 ± 1.55
65.0	4.54 ± 1.35	1.44 ± 0.50	6.35 ± 1.62
75.0	4.16 ± 1.22	1.40 ± 0.49	6.38 ± 1.66
85.0	3.94 ± 1.19	1.37 0.51	6.39 ± 1.70

Table S16: Kinetic data for ^{68}Ga-**Y2F**, derived from ROI analysis of 90 min dynamic PET scans (n = 3).

Time (min)	Blood	Muscle	Tumor
0.0	0.00 ± 0.00	0.00 ± 0.00	0.00 ± 0.00
0.1	4.42 ± 0.92	0.00 ± 0.00	0.13 ± 0.16
0.2	15.31 ± 0.79	0.00 ± 0.00	1.08 ± 0.43
0.3	25.08 ± 0.69	0.00 ± 0.00	2.04 ± 0.41
0.4	27.51 ± 2.57	0.15 ± 0.14	2.37 ± 0.22
0.5	25.75 ± 1.66	0.32 ± 0.42	2.68 ± 0.09
0.6	24.07 ± 2.09	1.04 ± 0.20	2.61 ± 0.24
0.8	22.36 ± 1.75	1.11 ± 0.17	2.55 ± 0.36
0.9	21.49 ± 1.36	1.30 ± 0.29	2.66 ± 0.35
1.1	20.30 ± 0.91	1.43 ± 0.24	2.82 ± 0.33
1.3	19.58 ± 0.87	1.43 ± 0.25	2.90 ± 0.31
1.4	19.23 ± 1.07	1.40 ± 0.30	3.00 ± 0.31
1.6	18.49 ± 0.98	1.44 ± 0.19	3.05 ± 0.44
1.8	18.11 ± 0.95	1.47 ± 0.28	3.07 ± 0.24
1.9	17.41 ± 0.89	1.46 ± 0.16	3.23 ± 0.39
2.1	17.15 ± 1.03	1.51 ± 0.22	3.31 ± 0.36
2.3	16.96 ± 1.13	1.54 ± 0.27	3.32 ± 0.44
2.4	16.51 ± 1.25	1.57 ± 0.08	3.43 ± 0.31
2.6	16.15 ± 1.25	1.79 ± 0.31	3.50 ± 0.26
2.8	15.96 ± 1.21	1.68 ± 0.54	3.61 ± 0.46
2.9	15.60 ± 1.33	1.55 ± 0.25	3.63 ± 0.31
3.1	15.36 ± 1.50	1.60 ± 0.15	3.63 ± 0.41
3.3	14.93 ± 1.43	1.76 ± 0.27	3.64 ± 0.34
3.4	14.83 ± 1.48	1.52 ± 0.17	3.86 ± 0.38
3.6	14.51 ± 1.53	1.71 ± 0.27	3.74 ± 0.46
3.8	14.22 ± 1.58	1.65 ± 0.33	3.89 ± 0.40
3.9	14.08 ± 1.43	1.59 ± 0.18	3.99 ± 0.39
5.0	12.61 ± 1.82	1.56 ± 0.02	4.22 ± 0.39
7.0	10.80 ± 2.03	1.62 ± 0.03	4.61 ± 0.44
9.0	9.63 ± 2.01	1.63 ± 0.05	4.91 ± 0.49
11.0	8.84 ± 2.02	1.63 ± 0.04	5.12 ± 0.48
13.0	8.26 ± 1.93	1.57 ± 0.02	5.30 ± 0.54
15.0	7.71 ± 1.77	1.61 ± 0.05	5.39 ± 0.61
17.0	7.26 ± 1.56	1.58 ± 0.02	5.52 ± 0.62
19.0	6.83 ± 1.34	1.56 ± 0.06	5.60 ± 0.65
22.5	6.28 ± 1.05	1.54 ± 0.04	5.74 ± 0.73
27.5	5.72 ± 0.68	1.48 ± 0.06	5.85 ± 0.79
32.5	5.44 ± 0.42	1.47 ± 0.07	5.93 ± 0.80
37.5	5.11 ± 0.29	1.43 ± 0.09	5.96 ± 0.87
42.5	4.90 ± 0.21	1.38 ± 0.08	5.99 ± 0.86
47.5	4.70 ± 0.17	1.36 ± 0.10	6.04 ± 0.88
52.5	4.46 ± 0.10	1.34 ± 0.10	6.05 ± 0.89
57.5	4.27 ± 0.14	1.29 ± 0.08	6.09 ± 0.91
65.0	3.99 ± 0.16	1.25 ± 0.09	6.12 ± 0.91
75.0	3.69 ± 0.20	1.18 ± 0.10	6.12 ± 0.98
85.0	3.48 ± 0.18	1.13 0.09	6.10 ± 1.00

Table S17: Kinetic data for ^{68}Ga-**Y3Y**, derived from ROI analysis of 90 min dynamic PET scans (n = 3).

Time (min)	Blood	Muscle	Tumor
0.0	0.00 ± 0.00	0.00 ± 0.00	0.00 ± 0.00
0.1	4.00 ± 3.37	0.00 ± 0.00	0.09 ± 0.15
0.2	12.05 ± 8.54	0.00 ± 0.00	0.50 ± 0.64
0.3	17.19 ± 10.42	0.00 ± 0.00	0.88 ± 0.97
0.4	20.19 ± 7.27	0.00 ± 0.00	1.11 ± 0.93
0.5	23.10 ± 3.56	0.00 ± 0.00	1.33 ± 0.77
0.6	21.19 ± 3.30	0.76 ± 0.14	1.42 ± 0.67
0.8	19.10 ± 1.93	0.90 ± 0.30	1.58 ± 0.70
0.9	20.22 ± 5.07	1.00 ± 0.22	1.73 ± 0.69
1.1	19.00 ± 4.17	1.08 ± 0.15	1.80 ± 0.58
1.3	17.50 ± 3.37	1.19 ± 0.17	1.88 ± 0.64
1.4	16.18 ± 2.29	1.24 ± 0.30	1.99 ± 0.68
1.6	15.36 ± 1.85	1.28 ± 0.20	2.10 ± 0.68
1.8	14.87 ± 1.74	1.29 ± 0.13	2.18 ± 0.64
1.9	14.39 ± 1.56	1.45 ± 0.20	2.26 ± 0.63
2.1	13.96 ± 1.51	1.49 ± 0.05	2.38 ± 0.63
2.3	13.45 ± 1.55	1.45 ± 0.14	2.39 ± 0.55
2.4	13.08 ± 1.24	1.41 ± 0.27	2.52 ± 0.53
2.6	12.62 ± 1.51	1.57 ± 0.19	2.56 ± 0.51
2.8	12.21 ± 1.39	1.62 ± 0.15	2.64 ± 0.44
2.9	11.94 ± 1.15	1.59 ± 0.20	2.70 ± 0.57
3.1	11.67 ± 1.30	1.60 ± 0.21	2.74 ± 0.49
3.3	11.42 ± 1.22	1.58 ± 0.31	2.77 ± 0.42
3.4	11.15 ± 1.21	1.60 ± 0.33	2.82 ± 0.43
3.6	10.93 ± 1.09	1.71 ± 0.13	2.94 ± 0.47
3.8	10.64 ± 1.18	1.60 ± 0.27	2.95 ± 0.38
3.9	10.55 ± 1.08	1.56 ± 0.18	3.06 ± 0.35
5.0	9.24 ± 1.09	1.61 ± 0.28	3.22 ± 0.34
7.0	7.65 ± 0.88	1.63 ± 0.24	3.51 ± 0.25
9.0	6.63 ± 0.83	1.63 ± 0.27	3.69 ± 0.22
11.0	5.93 ± 0.80	1.64 ± 0.27	3.81 ± 0.20
13.0	5.38 ± 0.75	1.59 ± 0.22	3.91 ± 0.19
15.0	4.96 ± 0.70	1.52 ± 0.24	3.96 ± 0.16
17.0	4.60 ± 0.70	1.53 ± 0.23	4.00 ± 0.17
19.0	4.30 ± 0.65	1.49 ± 0.18	4.02 ± 0.15
22.5	3.93 ± 0.62	1.44 ± 0.18	4.09 ± 0.14
27.5	3.49 ± 0.56	1.35 ± 0.17	4.11 ± 0.15
32.5	3.15 ± 0.51	1.29 ± 0.17	4.12 ± 0.14
37.5	2.89 ± 0.52	1.23 ± 0.17	4.11 ± 0.13
42.5	2.68 ± 0.47	1.16 ± 0.18	4.10 ± 0.14
47.5	2.49 ± 0.44	1.15 ± 0.14	4.10 ± 0.11
52.5	2.33 ± 0.46	1.10 ± 0.18	4.06 ± 0.14
57.5	2.16 ± 0.43	1.06 ± 0.15	4.05 ± 0.14
65.0	2.01 ± 0.40	0.99 ± 0.16	3.98 ± 0.18
75.0	1.82 ± 0.37	0.93 ± 0.17	3.91 ± 0.20
85.0	1.65 ± 0.34	0.89 0.15	3.84 ± 0.24

Table S18: Kinetic data for ^{68}Ga-**Y3F**, derived from ROI analysis of 90 min dynamic PET scans (n = 3).

Time (min)	Blood	Muscle	Tumor
0.0	0.85 ± 1.47	0.00 ± 0.00	0.00 ± 0.00
0.1	9.72 ± 5.35	0.00 ± 0.00	0.14 ± 0.16
0.2	18.84 ± 7.99	0.00 ± 0.00	0.64 ± 0.31
0.3	26.89 ± 7.33	0.01 ± 0.02	1.27 ± 0.20
0.4	29.52 ± 4.86	0.24 ± 0.42	1.71 ± 0.31
0.5	27.07 ± 3.36	0.46 ± 0.65	1.89 ± 0.52
0.6	21.14 ± 1.36	0.83 ± 0.10	1.84 ± 0.40
0.8	18.68 ± 0.81	1.16 ± 0.17	2.02 ± 0.42
0.9	17.58 ± 0.96	1.14 ± 0.06	2.18 ± 0.45
1.1	16.50 ± 1.08	1.23 ± 0.10	2.36 ± 0.40
1.3	15.68 ± 1.13	1.21 ± 0.17	2.47 ± 0.39
1.4	14.80 ± 1.05	1.59 ± 0.29	2.56 ± 0.34
1.6	14.22 ± 1.14	1.48 ± 0.65	2.65 ± 0.39
1.8	13.73 ± 1.07	1.57 ± 0.20	2.75 ± 0.43
1.9	13.28 ± 1.30	1.68 ± 0.32	2.85 ± 0.43
2.1	12.60 ± 1.28	1.49 ± 0.39	2.93 ± 0.38
2.3	12.14 ± 1.44	1.49 ± 0.28	3.03 ± 0.38
2.4	11.75 ± 1.30	1.50 ± 0.50	3.02 ± 0.34
2.6	11.58 ± 1.34	1.53 ± 0.23	3.27 ± 0.47
2.8	11.06 ± 1.25	1.41 ± 0.07	3.17 ± 0.38
2.9	10.74 ± 1.48	1.48 ± 0.10	3.26 ± 0.30
3.1	10.49 ± 1.25	1.51 ± 0.02	3.31 ± 0.29
3.3	10.20 ± 1.33	1.84 ± 0.35	3.37 ± 0.31
3.4	9.91 ± 1.32	1.61 ± 0.33	3.52 ± 0.39
3.6	9.68 ± 1.34	1.57 ± 0.49	3.48 ± 0.33
3.8	9.46 ± 1.33	1.84 ± 0.17	3.51 ± 0.25
3.9	9.14 ± 1.27	1.64 ± 0.18	3.62 ± 0.31
5.0	7.93 ± 1.18	1.67 ± 0.16	3.73 ± 0.20
7.0	6.35 ± 0.96	1.72 ± 0.22	4.03 ± 0.10
9.0	5.38 ± 0.85	1.69 ± 0.19	4.17 ± 0.05
11.0	4.72 ± 0.75	1.62 ± 0.07	4.30 ± 0.08
13.0	4.24 ± 0.71	1.62 ± 0.09	4.33 ± 0.17
15.0	3.89 ± 0.66	1.67 ± 0.11	4.40 ± 0.17
17.0	3.59 ± 0.61	1.56 ± 0.01	4.44 ± 0.23
19.0	3.37 ± 0.50	1.51 ± 0.06	4.45 ± 0.24
22.5	3.01 ± 0.47	1.47 ± 0.04	4.48 ± 0.28
27.5	2.65 ± 0.39	1.42 ± 0.03	4.46 ± 0.34
32.5	2.37 ± 0.34	1.36 ± 0.10	4.46 ± 0.36
37.5	2.14 ± 0.32	1.28 ± 0.12	4.42 ± 0.39
42.5	1.97 ± 0.30	1.21 ± 0.14	4.39 ± 0.41
47.5	1.82 ± 0.28	1.23 ± 0.10	4.37 ± 0.41
52.5	1.71 ± 0.26	1.19 ± 0.09	4.32 ± 0.40
57.5	1.60 ± 0.24	1.12 ± 0.18	4.28 ± 0.44
65.0	1.45 ± 0.25	1.09 ± 0.14	4.21 ± 0.44
75.0	1.30 ± 0.22	1.04 ± 0.17	4.13 ± 0.44
85.0	1.19 ± 0.22	0.97 0.18	4.03 ± 0.46

Table S19: Kinetic data for ^{68}Ga-**Y4**, derived from ROI analysis of 90 min dynamic PET scans (n = 3).

Time (min)	Blood	Muscle	Tumor
0.0	0.00 ± 0.00	0.00 ± 0.00	0.00 ± 0.00
0.1	3.33 ± 1.82	0.00 ± 0.00	0.01 ± 0.02
0.2	18.09 ± 4.10	0.00 ± 0.00	0.97 ± 0.87
0.3	29.77 ± 6.66	0.00 ± 0.00	1.98 ± 1.35
0.4	34.25 ± 5.16	0.58 ± 0.24	2.61 ± 1.68
0.5	30.05 ± 3.71	1.15 ± 0.29	3.02 ± 1.71
0.6	25.44 ± 1.65	1.24 ± 0.25	3.06 ± 1.41
0.8	22.62 ± 0.89	1.48 ± 0.15	3.24 ± 1.50
0.9	21.13 ± 0.65	1.51 ± 0.25	3.25 ± 1.16
1.1	19.86 ± 1.10	1.55 ± 0.23	2.96 ± 0.39
1.3	18.77 ± 0.83	1.51 ± 0.16	3.16 ± 0.64
1.4	18.24 ± 1.26	1.64 ± 0.12	3.09 ± 0.40
1.6	17.61 ± 1.30	1.66 ± 0.05	3.21 ± 0.40
1.8	17.08 ± 1.30	1.72 ± 0.19	3.27 ± 0.49
1.9	16.35 ± 1.56	1.72 ± 0.07	3.33 ± 0.47
2.1	16.02 ± 1.48	1.78 ± 0.11	3.37 ± 0.46
2.3	15.42 ± 1.59	1.75 ± 0.06	3.50 ± 0.48
2.4	15.29 ± 1.59	1.87 ± 0.02	3.49 ± 0.40
2.6	14.90 ± 1.76	1.87 ± 0.17	3.59 ± 0.41
2.8	14.51 ± 1.69	1.77 ± 0.06	3.60 ± 0.28
2.9	14.20 ± 1.85	1.87 ± 0.20	3.71 ± 0.32
3.1	13.82 ± 1.71	1.81 ± 0.14	3.75 ± 0.35
3.3	13.65 ± 1.88	1.89 ± 0.07	3.77 ± 0.32
3.4	13.30 ± 1.79	1.93 ± 0.09	3.88 ± 0.33
3.6	13.00 ± 1.75	1.95 ± 0.12	3.82 ± 0.31
3.8	12.70 ± 1.69	1.88 ± 0.18	3.81 ± 0.25
3.9	12.53 ± 1.81	2.06 ± 0.22	3.86 ± 0.29
5.0	11.13 ± 1.74	1.90 ± 0.09	4.00 ± 0.20
7.0	9.43 ± 1.74	1.87 ± 0.05	4.12 ± 0.17
9.0	8.21 ± 1.75	1.80 ± 0.09	4.18 ± 0.26
11.0	7.32 ± 1.76	1.77 ± 0.16	4.23 ± 0.30
13.0	6.61 ± 1.76	1.72 ± 0.14	4.20 ± 0.29
15.0	6.01 ± 1.66	1.68 ± 0.11	4.22 ± 0.31
17.0	5.59 ± 1.57	1.63 ± 0.08	4.16 ± 0.33
19.0	5.25 ± 1.51	1.59 ± 0.14	4.15 ± 0.35
22.5	4.75 ± 1.32	1.52 ± 0.13	4.08 ± 0.37
27.5	4.22 ± 1.08	1.42 ± 0.09	4.02 ± 0.39
32.5	3.82 ± 0.92	1.33 ± 0.11	3.94 ± 0.36
37.5	3.52 ± 0.80	1.26 ± 0.08	3.87 ± 0.39
42.5	3.24 ± 0.69	1.21 ± 0.07	3.79 ± 0.36
47.5	3.01 ± 0.62	1.15 ± 0.08	3.72 ± 0.40
52.5	2.81 ± 0.54	1.10 ± 0.06	3.68 ± 0.38
57.5	2.64 ± 0.47	1.05 ± 0.07	3.62 ± 0.40
65.0	2.43 ± 0.39	1.00 ± 0.06	3.54 ± 0.36
75.0	2.17 ± 0.30	0.92 ± 0.05	3.45 ± 0.38
85.0	1.94 ± 0.24	0.86 0.03	3.35 ± 0.36

Table S20: Kinetic data for ^{68}Ga-**Y6** (^{68}Ga-Trivehexin), derived from ROI analysis of 90 min dyn. PET scans (n = 3).

Time (min)	Blood	Muscle	Tumor
0.0	0.00 ± 0.00	0.00 ± 0.00	0.00 ± 0.00
0.1	3.40 ± 2.13	0.00 ± 0.00	0.16 ± 0.24
0.2	7.35 ± 4.77	0.26 ± 0.45	0.51 ± 0.60
0.3	11.96 ± 7.68	0.69 ± 1.20	0.87 ± 0.92
0.4	17.23 ± 11.03	0.61 ± 1.05	1.45 ± 1.19
0.5	19.30 ± 10.77	1.00 ± 1.72	1.80 ± 1.18
0.6	20.51 ± 10.61	1.15 ± 1.26	1.99 ± 1.07
0.8	19.30 ± 4.45	1.19 ± 1.01	2.45 ± 1.03
0.9	20.40 ± 2.40	1.52 ± 1.24	2.85 ± 0.83
1.1	18.82 ± 2.36	1.59 ± 1.15	2.98 ± 0.91
1.3	17.62 ± 2.21	1.83 ± 1.09	3.26 ± 0.82
1.4	16.65 ± 2.12	1.82 ± 0.98	3.37 ± 0.77
1.6	16.00 ± 2.12	2.07 ± 1.35	3.50 ± 0.78
1.8	15.43 ± 2.26	1.90 ± 1.41	3.70 ± 0.75
1.9	14.84 ± 2.05	2.01 ± 1.15	3.68 ± 0.80
2.1	14.30 ± 2.01	1.92 ± 1.14	3.99 ± 0.80
2.3	13.77 ± 1.90	1.95 ± 0.94	4.06 ± 0.85
2.4	13.40 ± 2.08	1.90 ± 1.06	4.16 ± 0.85
2.6	13.14 ± 1.99	1.93 ± 0.91	4.31 ± 0.80
2.8	12.75 ± 1.84	2.00 ± 1.00	4.40 ± 0.77
2.9	12.54 ± 1.97	2.34 ± 1.17	4.46 ± 0.66
3.1	12.04 ± 1.74	2.12 ± 1.26	4.48 ± 0.80
3.3	11.94 ± 1.93	2.17 ± 1.19	4.70 ± 0.83
3.4	11.44 ± 1.87	1.81 ± 0.97	4.75 ± 0.74
3.6	11.26 ± 1.76	2.33 ± 1.46	4.61 ± 0.80
3.8	10.99 ± 1.86	2.06 ± 1.24	4.80 ± 0.65
3.9	10.79 ± 1.86	2.30 ± 1.00	4.88 ± 0.72
5.0	9.54 ± 1.67	2.12 ± 1.06	5.14 ± 0.73
7.0	7.89 ± 1.29	2.16 ± 0.92	5.60 ± 0.62
9.0	6.86 ± 1.16	2.05 ± 0.79	5.87 ± 0.63
11.0	6.09 ± 1.01	2.02 ± 0.75	6.07 ± 0.61
13.0	5.56 ± 0.88	1.99 ± 0.62	6.20 ± 0.62
15.0	5.12 ± 0.85	1.94 ± 0.65	6.28 ± 0.68
17.0	4.78 ± 0.79	1.89 ± 0.63	6.37 ± 0.72
19.0	4.49 ± 0.75	1.81 ± 0.51	6.38 ± 0.75
22.5	4.10 ± 0.69	1.77 ± 0.51	6.47 ± 0.80
27.5	3.66 ± 0.64	1.66 ± 0.43	6.49 ± 0.84
32.5	3.32 ± 0.59	1.57 ± 0.39	6.50 ± 0.92
37.5	3.06 ± 0.53	1.50 ± 0.35	6.46 ± 0.97
42.5	2.85 ± 0.52	1.46 ± 0.31	6.41 ± 0.99
47.5	2.67 ± 0.50	1.40 ± 0.31	6.34 ± 1.02
52.5	2.53 ± 0.49	1.34 ± 0.32	6.31 ± 1.07
57.5	2.40 ± 0.48	1.34 ± 0.29	6.24 ± 1.11
65.0	2.26 ± 0.49	1.26 ± 0.28	6.17 ± 1.13
75.0	2.11 ± 0.50	1.20 ± 0.26	6.05 ± 1.17
85.0	1.99 ± 0.54	1.14 0.24	5.93 ± 1.21
